# Obesity and response to anti-tumor necrosis factor-α agents in patients with select immune-mediated inflammatory diseases: A systematic review and meta-analysis

**DOI:** 10.1371/journal.pone.0195123

**Published:** 2018-05-17

**Authors:** Siddharth Singh, Antonio Facciorusso, Abha G. Singh, Niels Vande Casteele, Amir Zarrinpar, Larry J. Prokop, Eduardo L. Grunvald, Jeffrey R. Curtis, William J. Sandborn

**Affiliations:** 1 Division of Gastroenterology, University of California San Diego, La Jolla, California, United States of America; 2 Division of Biomedical Informatics, University of California San Diego, La Jolla, California, United States of America; 3 Gastroenterology Unit, Department of Medical Sciences, University of Foggia, Foggia, Italy; 4 Division of Rheumatology, Allergy and Immunology, University of California San Diego, La Jolla, California, United States of America; 5 Institute for Diabetes and Metabolic Health, University of California, San Diego, La Jolla, California, United States of America; 6 VA San Diego Health Systems, La Jolla, California, United States of America; 7 Department of Library Services, Mayo Clinic, Rochester, Minnesota, United States of America; 8 Weight Management Program, Department of Medicine, University of California San Diego, La Jolla, California, United States of America; 9 Division of Clinical Immunology and Rheumatology, University of Alabama at Birmingham, Birmingham, Alabama, United States of America; VU University Medical Center, NETHERLANDS

## Abstract

**Objectives:**

We sought to evaluate the association between obesity and response to anti-tumor necrosis factor-α (TNF) agents, through a systematic review and meta-analysis.

**Methods:**

Through a systematic search through January 24, 2017, we identified randomized controlled trials (RCTs) or observational studies in adults with select immune-mediated inflammatory diseases–inflammatory bowel diseases (IBD), rheumatoid arthritis (RA), spondyloarthropathies (SpA), psoriasis and psoriatic arthritis (PsA)–treated with anti-TNF agents, and reporting outcomes, stratified by body mass index (BMI) categories or weight. Primary outcome was failure to achieve clinical remission or response or treatment modification. We performed random effects meta-analysis and estimated odds ratios (OR) and 95% confidence interval (CI).

**Results:**

Based on 54 cohorts including 19,372 patients (23% obese), patients with obesity had 60% higher odds of failing therapy (OR,1.60; 95% CI,1.39–1.83;I^2^ = 71%). Dose-response relationship was observed (obese vs. normal BMI: OR,1.87 [1.39–2.52]; overweight vs. normal BMI: OR,1.38 [1.11–1.74],p = 0.11); a 1kg/m^2^ increase in BMI was associated with 6.5% higher odds of failure (OR,1.065 [1.043–1.087]). These effects were observed across patients with rheumatic diseases, but not observed in patients with IBD. Effect was consistent based on dosing regimen/route, study design, exposure definition, and outcome measures. Less than 10% eligible RCTs reported outcomes stratified by BMI.

**Conclusions:**

Obesity is an under-reported predictor of inferior response to anti-TNF agents in patients with select immune-mediated inflammatory diseases. A thorough evaluation of obesity as an effect modifier in clinical trials is warranted, and intentional weight loss may serve as adjunctive treatment in patients with obesity failing anti-TNF therapy.

## Introduction

The global prevalence of obesity is rising, with one in 10 people across the world being classified as obese.[1, 2] In the United States, over 35% adults are obese, and increased healthcare spending on this population is estimated to account for nearly a third of the growth in healthcare expenditure.[3] Obesity may contribute to increased risk of developing select immune-mediated inflammatory diseases (IMIDs) such as rheumatoid arthritis (RA), psoriasis and Crohn’s disease (CD), and approximately 10–50% of patients with IMIDs are obese.[4–9] Obesity has been associated with more severe disease activity, inferior quality of life and higher burden of hospitalization in patients with these immune-mediated diseases.[5, 6, 10–14]

Targeted immunomodulators such as anti-tumor necrosis factor-α (TNF), are the mainstay of therapy for patients with select IMIDs with moderate-severe disease. Clinical response to these agents is seen in 40–80% patients with select IMIDs.[15–19] Population pharmacokinetic studies of different anti-TNF agents have consistently shown that high body weight is associated with accelerated clearance, resulting in lower trough concentrations.[20–22] Additionally, obesity, particularly visceral fat, independently contributes to higher systemic inflammatory burden.[23, 24] Several observational studies have shown that obesity may be a negative prognostic marker in patients with rheumatic diseases,[11, 25] and variably and inconsistently shown an inferior response to biologic agents in obese patients.[26–33]

Hence, we sought to systematically review the association between obesity and response to anti-TNF agents across selected IMIDs, and examine whether the effect varies across different diseases and between different anti-TNF agents based on route of administration (subcutaneous vs. intravenous) and dosing scheme (weight-based vs. fixed dosing).

## Methods

This systematic review is reported according to the Preferred Reporting Items for Systematic Reviews and Meta-Analyses (PRISMA) Statement and was conducted following *a priori* established protocol (S1 Text).[34]

### Selection criteria

We included phase 2 or phase 3 randomized controlled trials (RCT) or observational cohort studies, with at least 2 months follow-up, that met the following inclusion criteria: (1) patients with select IMIDs (IBD including CD or ulcerative colitis [UC], RA, psoriasis or psoriatic arthritis [PsA], spondyloarthropathy [SpA]), treated with US Food and Drug Administration (FDA)-approved anti-TNF agents (infliximab, adalimumab, certolizumab pegol, golimumab, etanercept), (b) outcome reported as failure to achieve clinical remission or response or need for treatment modification, and (c) outcomes stratified by baseline body mass index (BMI) or body weight. For our analysis, RCTs were considered as prospective cohort studies, and only patients receiving active intervention with anti-TNF agents were included in analysis; if two or more arms in the RCTs were receiving different active biologic therapy, then they were considered separate cohorts. We excluded cross-sectional studies or case-control studies, studies of non-TNF biologic agents, immunomodulators or small molecules, or studies in which outcomes were not stratified by baseline BMI or weight. We also excluded studies in which the exposure categories did not include any patients with overweight or obesity patients (for example, BMI <20 vs. ≥20kg/m^2^).[35] When there were multiple publications from the same cohort, we only included data from the most recent comprehensive report.

### Data sources, search strategy and study selection

The search strategy was designed and conducted by an experienced medical librarian with input from study investigators, utilizing various databases from inception to January 24, 2017. The databases included Ovid Medline (January 1, 1946 to January 24, 2017), EMBASE (January 1, 1988 to January 24, 2017), Scopus (January 1, 2004 to January 24, 2017), Web of Science (January 1, 1990 to January 24, 2017), and Cochrane Central Register of Controlled Trials (January 1, 2005 to January 24, 2017). In addition, we searched clinical trial registries (www.clinicaltrials.gov and www.clinicaltrialsregister.eu), conference proceedings and published systematic reviews for additional studies. Controlled vocabulary supplemented with keywords was used to search for observational studies (S2 Text) and RCTs (S3 Text) reporting impact of obesity on response to anti-TNF therapy. Two authors independently reviewed the title and abstract of studies identified in the search to exclude studies that did not answer the research question of interest, based on pre-specified inclusion and exclusion criteria. The full text of the remaining articles was independently reviewed, to determine whether it contained relevant information. Next, we manually searched the bibliographies of the selected articles, as well as review articles on the topic for additional articles. We also performed a manual search of conference proceedings from major gastroenterology, rheumatology and dermatology meetings (Digestive Diseases Week, Annual Meeting of the American College of Rheumatology, Annual European Congress of Rheumatology, and Annual meeting of the American College of Dermatology from 2012 to 2016) for additional abstracts on the topic. Since RCTs may report results stratified by body weight or BMI in subgroup analyses in online supplements alone, we reviewed full-texts of all published phase 2 and 3 RCTs of candidate anti-TNF agents in detail, and on clinical trial registry websites (www.clinicaltrials.gov, www.clinicaltrialsregister.eu).

### Data abstraction and risk of bias assessment

After study selection, two authors independently abstracted data on study and patient characteristics, exposure variables, outcomes, confounding variables and statistical analyses, using a standardized data abstraction form. Details of data abstraction are shown in the appendix (S4 Text).

Risk of bias was assessed by 2 investigators independently, using the Quality In Prognosis Studies tool, which evaluates validity and bias in studies of prognostic factors across six domains: participation, attrition, prognostic factor measurement, confounding measurement and account, outcome measurement, and analysis and reporting.[36]

### Outcomes assessed

The primary outcome of interest was failure of index biologic therapy. This was defined using a hierarchy of the following outcomes, i.e., if the first outcome was not reported, then the next reported outcome of interested was chosen: (1) failure to achieve clinical remission, (2) failure to achieve clinical response (based on validated disease activity indices or author-defined criteria); or (3) need for treatment modification (discontinuation or escalation of index therapy due to ineffectiveness and/or switching to alternative therapy, and/or need for surgery).

In order to evaluate stability of the association between obesity and response to anti-TNF therapy, and examine potential sources of heterogeneity, we performed several *a priori* subgroup analyses based on: type of diseases (IBD vs. RA vs. SpA vs. psoriasis-PsA), route of administration (subcutaneous vs. intravenous), dosing scheme (weight-based dosing vs. fixed dosing), exposure categories (based on BMI: obese vs. normal BMI, obese vs. non-obese [BMI≥30kg/m^2^ vs. BMI<30kg/m^2^], overweight vs. non-overweight [BMI≥25kg/m^2^ vs. BMI<25kg/m^2^]), outcome definition (based on disease activity indices vs. need for modification of therapy) and study design (RCTs vs. cohort). Sensitivity analysis restricting to studies that adjusted for key confounding variables was performed. We also performed meta-regression to assess whether effect estimates varied depending on prevalence of exposure (proportion of obese patients) or the prevalence of the outcome (proportion who failed index biologic therapy).

We examined dose-response relationship using two approaches. First, we limited analysis to studies reporting the association between 2 or more categories of obesity against a reference category, and compared as nominal groups. For example, for studies using WHO-defined categories, then comparing obese vs. normal BMI and overweight vs. normal BMI; for studies reporting tertiles of BMI or weight, then comparing 3^rd^ tertile (T_3_) vs. 1^st^ tertile (T_1_) and 2^nd^ tertile (T_2_) vs. 1^st^ tertile (T_1_). When studies reported multiple different exposure categories, then all categories beyond the bottom two were collapsed into a single category (labeled high BMI or weight) for consistent comparison. Second, studies that reported analyses per 1kg/m^2^ change in BMI were pooled separately.

### Statistical analysis

We used the random-effects model described by DerSimonian and Laird to calculate summary OR and 95% confidence intervals (CI).[37] Maximally adjusted OR, where reported in studies, was used for analysis to account for confounding variables; where not reported, unadjusted OR were used or calculated based on event rates in different exposure categories. To estimate what proportion of total variation across studies was due to heterogeneity rather than chance, I^2^ statistic was calculated.[38] In this, a value of <30%, 30%-60%, 60%-75% and >75% were suggestive of low, moderate, substantial and considerable heterogeneity, respectively. Between-study sources of heterogeneity were investigated using subgroup analyses by stratifying original estimates according to study characteristics (as described above). In this analysis, a p-value for differences between subgroups of <0.10 was considered statistically significant. Publication bias was assessed qualitatively using funnel plots and quantitatively using Egger’s regression test.[39]

All analyses were performed using Comprehensive Meta-Analysis version 2.0 (Englewood, New Jersey). Since this was a meta-analysis of published studies, institutional review board approval was not required.

## Results

**Fig 1:**
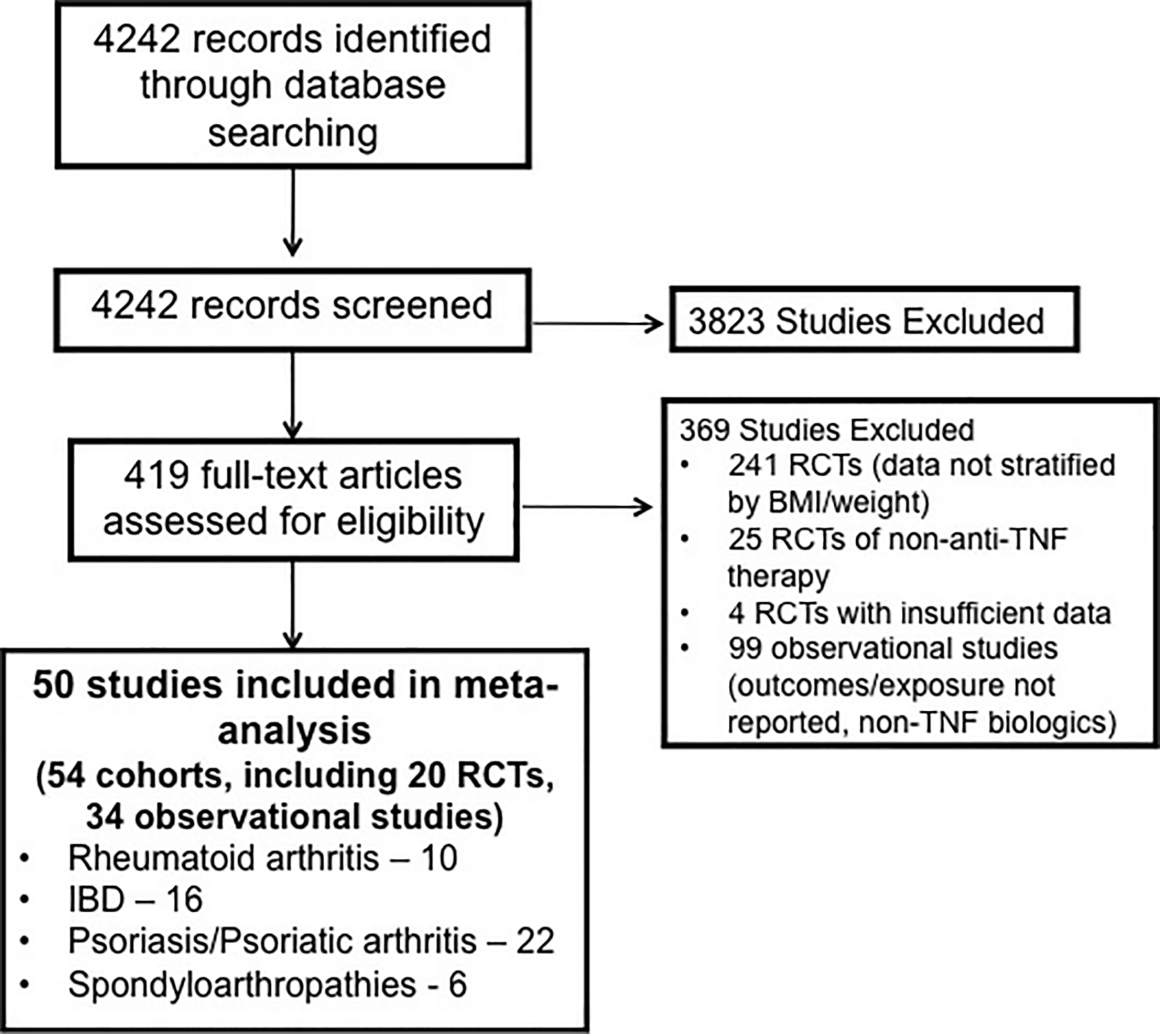
Study selection flowsheet.

From 4242 unique studies identified based on systematic review, 3823 articles were excluded based on title and abstract review (since these were unrelated to biologic therapies or unrelated to diseases of interest). Overall, 419 articles were reviewed in full for eligibility. Of 241 eligible RCTs reviewed in full, 20 (8.3%) reported results stratified by BMI or weight and were included; the remainder did not provide data stratified by BMI or weight. Twenty five RCTs of non-TNF biologics were excluded, and 5 RCTs were excluded since the outcomes of interest were not reported. Of 149 cohort studies, 99 were excluded since they included either non-TNF biologics (n = 37), did not adequately stratify results by BMI or weight (n = 46) or were unrelated to diseases of interest (n = 16). We included 54 cohorts derived from 50 studies for categorical analysis,[14, 26–33, 40–80] and 9 cohorts from 8 studies for per-unit dose-response analysis.[32, 33, 42, 54, 81–84] These included 34 observational cohorts (n = 13,336) and 20 cohorts derived from RCTs (n = 6,036). Fig 1 shows the study selection flowchart.

### Characteristics of included studies

Tables 1 and 2 provide details of included studies and participants. Overall, these 54 cohorts reported on 19,372 patients; 10, 16, 6 and 22 studies included patients with RA, IBD, SpA and psoriasis-PsA, respectively. Median 23% (IQR, 16–31.5%) patients were classified as obese in these studies. Follow-up ranged from 2–36 months. Overall, the studies were deemed to be at moderate risk of bias, due to failure to adjust for confounding variables and non-standard exposure definition (S1 Table).

**Table 1:** Characteristics of included studies–RCTs.

Author, Year of publication	Design; Intervention (N), Comparator (N)	BMI in biologic intervention arm; exposure categories	Outcome; timing of measurement; failure to achieve outcome (%)	Patient characteristics (Age [SD], % males)	Concomitant therapy
**RHEUMATOID ARTHRITIS**					
Smolen, 2011 [75] (open-label enrollment into PRESERVE) [ABSTRACT]	Open-label; ETN 50mg SQ + MTX qw; 36 weeks (N = 761); no comparator	BMI<25–386 (50.7%); BMI 25–29–248 (32.6%) BMI≥30–127 (16.7%)	Failure to achieve remission (DAS28<2.6), 36 weeks; 32.5%	Age: 48 (12) Sex: 17%	CS– 59%; IM (MTX)– 100%
Kaeley, 2016[57] (MUSICA) [ABSTRACT]	ADA 40mg SQ qow + MTX 20mg qw (N = 155) vs. ADA 40mQ SW qow + MTX 7.5mg qw (N = 154)	BMI<25–69 (22.3%); BMI 25–29–102 (33.0%) BMI≥30–137 (44.3%)	Failure to achieve response (ACR50), 24 weeks; 65.2%	Age: 55 (12) Sex: 25%	CS– 42%; IM (MTX)– 100%
Heimans, 2013[54] (BeSt study)	4 arms (only arm 4 included for this analysis): Group 1. Sequential monotherapy starting with MTX (N = 126); Group 2: step-up combination therapy starting with MTX (N = 121), Group 3: initial combination therapy,with the COBRA scheme: MTX, sulfasalazine and tapered highdose prednisone (N = 133), vs. Group 4: a combination of MTX and IFX 3mg/kg IV every 8 weeks (N = 128)	Only group 4: Not reported Overall, BMI<25–216 (42.5%) BMI≥25–292 (57.5%)	Failure to achieve remission (DAS28<2.6), 52 weeks; NR (Group 4: RR of failing to achieve remission in patients with BMI≥25 vs. BMI<25 = 2.20 [0.99–4.92]	Age: 54 (14) Sex: 34%	NR
Weinblatt, 2013[79] (GO-FURTHER)	Golimumab 2mg/kg IV at w0 and w4, and then q8w (n = 395) + MTX vs. placebo + MTX	Weight categories: <59.6 kg: 95 59.6–69.6 kg: 100 69.6–81.15 kg: 101 >81.15 kg: 98	Failure to achieve response (ACR20), 14 weeks; 41%	Age: 52 (13) Sex: 18%	CS–NR IM (MTX): 100%
**INFLAMMATORY BOWEL DISEASES**					
Kobayashi, 2016[58]	IFX 5mg/kg IV at weeks 0,2,6 and then q8w (N = 104) vs. placebo (N = 104) (Ulcerative colitis)	Weight, mean (SD): 57.6 (12.7); Exposure categories: Weight <56kg vs. ≥56kg	Failure to achieve remission (CAI≤4), week 14; 44%	Age: 40 (13) Sex: 64%	CS– 65% IM– 48%
Sandborn, 2012[73]; (ULTRA 2)	ADA 160/80, and then 40mg SQ qow (N = 248) vs. placebo (N = 246) (Ulcerative colitis)	Weight, mean (SD): 75.3 (17.7); Exposure categories: Weight <70kg vs. ≥70kg	Failure to achieve remission (MCS<3), week 52; 82.7%	Age: 40 (12) Sex: 57%	CS– 61% IM– 38%
Sandborn, 2011 [72]	CZP 400mg SQ at weeks 0,2,4 (N = 223) vs. Placebo (N = 215) (Crohn’s disease)	BMI<25–124 (55.6%) BMI≥25–91 (44.4%)	Failure to achieve remission (CDAI<150), week 6; 68%	Age: 36 (13) Sex: 47%	CS– 44% IM– 35%
Reinisch, 2011[69] (ULTRA 1)	ADA 160/80, and then 40mg SQ qow (N = 130) vs. placebo (N = 130) (Ulcerative colitis)	Weight, mean (SD): 75.5 (14.2); Exposure categories: Weight <70kg vs. 70–81kg vs. ≥82kg	Failure to achieve remission (MCS<3), week 8; 81.5%	Age: 36 (14) Sex: 64%	CS– 37% IM– 22%
Colombel, 2010 [48] (SONIC)	IFX 5mg/kg IV at weeks 0,2,6 and then q8w (N = 169) vs. IFX + Azathioprine 2.5mg/kg po (N = 169) vs. Azathioprine (N = 170) (Crohn’s disease)	Weight, median (Groups 1 and 2): 68.9kg, 69.6kg; Exposure categories: Weight <60kg vs. 60–74kg vs. ≥75kg	Failure to achieve remission (CDAI<150); week 26; 49.4%	Age, median: 35 Sex: 51%	CS– 29% IM– 50%
Sandborn, 2009[71] (ACT-1 and ACT-2 trials)	IFX 5mg/kg or 10mg/kg IV at weeks 0,2,6, and then every 8 weeks (N = 448) vs. placebo (N = 244) (Ulcerative colitis)	ACT1 Weight, mean (SD): IFX 5mg/kg: 80.0 (17.8), IFX 10mg/kg: 76.9 (17.1); ACT2 Weight, mean (SD): IFX 5mg/kg: 78.4 (17.8), IFX 10mg/kg: 79.6 (20.6); Exposure category: Weight <76kg vs. ≥76kg	Colectomy, week 54; 10.3% (variables adjusted for age, sex, disease duration, disease extent, baseline disease severity, concomitant medications, CRP)	ACT1: Age: 42 (15) Sex: 62% ACT2: Age: 40 (13) Sex: 60%	CS: 56% IM: 47%
**PSORIASIS-PSORIATIC ARTHRITIS**					
Cai, 2017[44]	ADA 80mg then 40mg SQ qow (N = 338) vs. Placebo (N = 87) (Psoriasis)	BMI<25–193 (51.4%); BMI 25–29–126 (37.3%); BMI≥30–19 (5.6%); Exposure categories: BMI <25 vs. ≥25	Failure to achieve response (PASI75); week 12; 22.2%	Age: 43 (12) Sex: 75%	None
Prussick, 2015[67] (CHAMPION trial)	ADA 80mg then 40mg SQ qow (N = 99) vs. MTX (N = 94) vs. Placebo (N = 48) (Psoriasis)	BMI<25–40 (40.4%); BMI 25–29–28 (28.3%) BMI≥30–31 (31.3%)	Failure to achieve response (PASI75), 16 weeks; 22.2%	Normal vs. overweight vs. obese Age: 37 (11) vs. 47 (14) vs. 44 (12) Sex: 53% vs. 82% vs. 73%	None
Poulin, 2014[66] (REACH trial)	ADA 80mg then 40mg SQ qow (N = 49) vs. Placebo (N = 23) (Psoriasis)	Weight, mean (SD): 90.4 (19.7); Exposure categories: Weight <88kg vs. ≥88kg	Failure to achieve ‘clear’ or ‘almost clear’ on hfPGA scale; week 16; 69.4%	Age: 49 (11) Sex: 43%	None
Gottlieb, 2012[52]	ETN 50mg SQ twice/week x 12 weeks, then 50mg qw + MTX (N = 239) vs. ETN 50mg SQ twice/week x 12 weeks, then 50mg qw + placebo (N = 239) (Psoriasis)	BMI, mean (SD): 32.3 (7.5); Exposure categories: BMI ≤35 vs. >35	Failure to achieve response (PASI75); week 24; 31.2%	Age: 45 (13) Sex: 67%	None
Bagel, 2012[41]	ETN 50mg SQ twice/week x 12 weeks, then 50mg qw + placebo qw x 12 weeks (N = 62) [Cohort 1] vs. Placebo x 12 weeks, then ETN 50mg SQ twice/week x 12 weeks (N = 62) [Cohort 2] (Psoriasis)	BMI, median (range): 30.2 (18.2–44.2) [Cohort 1]; 30.1 (19.1–49.5); Exposure categories: BMI ≤35 vs. >35	Failure to achieve response (PASI75); week 12 for cohort 1; week 24 for cohort 2; 40.3%	Cohort 1 Age: 39 (13) Sex: 53% Cohort 2 Age: 42 (13) Sex: 58%	None
Paul, 2012[65](BELIEVE trial)	ADA 80mg then 40mg SQ qow + topical calcipotriol/betamethasone dipropionate vs. ADA+matching topical vehicle (N = 730) (Psoriasis and psoriatic arthritis)	Weight, mean (SD): 84.6 (19); Exposure categories: Weight quartiles	Failure to achieve response (PASI75), 16 weeks; 32.9%	Age>40: 66.6% Sex: 69%	None
Menter, 2010[60] (REVEAL trial)	ADA 80mg then 40mg SQ qow (N = 814) vs. Placebo (N = 398) (Psoriasis)	BMI<25–144 (17.7%); BMI 25–29–273 (33.5%) BMI≥30–395 (48.5%)	Failure to achieve response (PASI75), week 16; 29%	Age: 44 (13) Sex: 67%	None
**SPONDYLOARTHRITIS**					
Huang, 2014[56]	ADA 40mg SQ qow (N = 229) vs. Placebo (N = 115)	Weight, mean (SD): 63.3 (12.4); Exposure categories: Weight <60kg vs. ≥60kg	Failure to achieve response (ASAS20), week 12; 32.8%	Age: 30 (9) Sex: 81%	CS: 4% IM: 23%
Mease, 2014[59] (ABILITY-1 trial)	ADA 40mg SQ qow (N = 91) vs. Placebo (N = 94)	BMI<25–42 (46.2%) BMI 25–29–37 (40.6%) BMI≥30–12 (13.2%)	Failure to achieve response (ASAS20), week 12; 48.3%	Age: 38 (11) Sex: 52%	None

ADA = Adalimumab; ASAS = Assessment of Spondyloarthritis International Society; BMI = Body mass index; CAI = Clinical activity index; CDAI = Crohn’s disease activity index; CS = corticosteroids; CZP = Certolizumab pegol; ETN = Etanercept; hfPGA = hand/feet physician global assessment; IFX = Infliximab; IM = Immunomodulators; MTX = Methotrexate; PASI = Psoriasis Area Severity Index; qw = every week; qow = every other week; SD = standard deviation

**Table 2:** Characteristics of included studies–observational studies.

Author, Year of publication	Location, Time Period; Study Design	Medications	BMI (Mean, SD) and Exposure categories	Outcome; timing of measurement; failure to achieve outcome (%)	Patient characteristics (Age, % males, % concomitant steroids)
**RHEUMATOID ARTHRITIS**					
Iannone, 2015 [27]	Italy, 2003–14; Retrospective	ADA: 68; ETN: 147; IFX: 73; CZP: 4	BMI<25–117 (40.1%) BMI 25–29–109 (37.3%) BMI≥30–66 (22.6%)	Failure to achieve remission (ESR-DAS28<2.6), 12m; NR	Normal vs. overweight vs. obese Age: 54 (20) vs. 61 (14) vs. 61 (14) Sex: 10% vs. 18% vs. 17% Steroids: 70% vs. 85% vs. 73%
Ottaviani, 2015[29]	France, 2005–12; Retrospective	IFX: 76	BMI<25–25 (32.9%) BMI 25–29–29 (38.2%) BMI≥30–22 (28.9%); Exposure categories: BMI ≥30 vs. <30	Failure to achieve remission (DAS28<2.6), 6m; 46.1% (variables adjusted for: age, sex, disease duration, ESR, CRP, prior anti-TNF therapies, concomitant immunomodulator therapies, baseline disease activity, RF and CCP status)	Age, median (IQR): 49 (42–56) Sex: 17% Steroids: 93%
Rodrigues, 2014[70][Abstract]	Portugal, NR; Retrospective	Anti-TNF: 317	BMI<30–244 (67%) BMI≥30–73 (23%)	Failure to achieve remission (DAS28<2.6), 6m; NR (variables adjusted for: NR)	NR
Gremese, 2013[32]	Italy, NR; Retrospective	ADA: 260; ETN: 227; IFX: 154	BMI<25–368 (57.4%) BMI 25–29–207 (32.3%) BMI≥30–66 (10.3%)	Failure to achieve remission (DAS28<2.6); 12m; 69.7%; adjusted OR (per unit BMI) for failure to achieve remission: 1.12 [1.01–1.24] (variables adjusted for: age, sex, disease duration, DAS28, ESR, VAS pain, global health, HAQ, steroids, RF, anti-CCP, drug)	Age: 52 (14) Sex: 19% Steroids: 38.9%
Klaasen, 2011[30]	Holland, NR; Retrospective	IFX: 89	BMI<20–8 (9.0%) BMI 20–29–66 (74.2%) BMI≥30–15 (16.8%)	Failure to achieve response (ΔDAS28<1.2), 16 weeks; 28%	BMI<20 vs. BMI 20–30 vs. BMI>30 Age: 50 (15) vs. 57 (11) vs. 53 (15) Sex: 25% vs. 29% vs. 13% Steroids: 25% vs. 33% vs. 20%
Abhishek, 2010[40]	UK, 2001–08; Retrospective	Anti-TNF: 395	BMI, mean (SD)– 27.0 (6.4); Exposure categories: BMI tertiles, T1, ≤23.5 vs. T2, 23.6–28.6 vs. T3, >28.6	Failure to achieve at least moderate EULAR response, 3m; 10.6% (variables adjusted for: age, sex, disease duration, prior DMARDs, DAS28, concurrent MTX or prednisone, smoking, RF, other comorbidities)	Age: 61 (12) Sex: 23% Steroids: 31.6%
**INFLAMMATORY BOWEL DISEASES**					
Guerbau, 2016[53] [Abstract]	France, 2009–14; Retrospective	IFX: 140 (CD)	BMI<25–96 (68.6%) BMI 25–29–21 (15.0%) BMI≥30–23 (16.4%)	Rate of optimization of infliximab therapy; 12m; median delay in infliximab dose escalation was shorter in obese and overweight patients vs. normal BMI patients: 8.5m vs. 8m vs. 17m (p = 0.03)	No significant differences in baseline demographic and clinical characteristics of patients in 3 BMI categories (disease location/behavior, disease activity, concomitant immunosuppressives, C-reactive protein)
Brown, 2016[43]	UK, 1999–2012; Retrospective	IFX: 388 (CD)	BMI<18.5–33 (10.4%) BMI 18.5–24.9–218 (56.2%) BMI 25–29–91 (23.5%) BMI≥30–46 (11.9%) BMI≥30 vs. BMI 25–30 vs. BMI 18.5–24.9	Loss of response (any of: dose escalation, switching to alternative agent, need for steroids, hospitalization related to CD, CD- related surgery), 12m; 41.6% (variables adjusted for: sex, disease localization, thiopurine or steroids use, perianal disease, age, duration of disease)	Age: 37 (14) Sex: 46% Steroids: 35.1%
Billiet, 2016[81]	Belgium, 1994–2016; Retrospective	IFX: 261 (CD)	BMI, median (IQR): 22.1 (19.4–24.7); Exposure categorized: *per unit BMI*	IFX failure free survival (loss of response, persistent and high-titer anti-drug antibodies, need for surgery); IFX-failure free survival at 12m, 93.7%; per unit BMI: HR, 1.06 [1.01–1.13], i.e., 6% higher risk of treatment failure per unit increase in BMI (only significant on univariate analysis)	Age: 31 (22–43) Sex: 47% Steroids: 21.5%
Harper, 2013[33]	USA, 2008–11; Retrospective	IFX: 123 (99 CD, 24 UC)	BMI, mean (SD): CD– 26.5 (6); UC– 26.4 (7.4); Exposure categories: BMI≥30 vs. BMI<30	Time to clinical flare (any of: dose escalation, switching to alternative agent, need for steroids, hospitalization related to IBD, IBD- related surgery); HR for time to clinical flare per unit BMI: CD– 1.06 [1.01–1.11], UC– 1.30 [1.07–1.58] (variables adjusted for: prior surgery, steroid use, extraintestinal manifestations, age, disease duration)	Age: CD– 35 (13); UC– 35 (15) Sex: CD– 48%; UC– 53% Steroids: CD– 35.4%; UC– 66.7%
Bhalme, 2013[42]	UK, 2000–09; Retrospective	IFX: 76; ADA: 54 (CD)	BMI, mean (SD): IFX– 24.5 (6); ADA– 25.7 (5.8); BMI<30: IFX– 62; ADA– 46 BMI≥30: IFX– 14; ADA– 8 Exposure categories: BMI≥30 vs. BMI<30	Time to loss of response (need for escalation of therapy); 12m; Proportion of patients requiring escalation of therapy at 1 year: BMI 25 vs. 30 vs. 35: IFX– 19% vs. 16% vs. 15%; ADA– 20% vs. 29% vs. 40%	Age: IFX– 39 (15); ADA– 40 (16) Sex: IFX– 45%; ADA– 33% Steroids: NR
Rosen, 2012[84] [ABSTRACT]	USA, 2002–11; Prospective	CZP: 74 (CD)	Exposure categories: per unit BMI	Need for surgery; 12m; adjusted OR for need for surgery per unit increase in BMI– 1.14 [1.01–1.30] (variables adjusted for: ESR)	Age: 36 (14) Sex: 28% Steroids: NR
Horst, 2012[55] [ABSTRACT]	USA, 2008–11; Retrospective	CZP: 107 (CD)	BMI≤25–44 BMI>25–63	Discontinuation of therapy, NR (variables adjusted for: age, sex, smoking, disease type)	Age: 39 (14) Sex: 38% Steroids: NR
Click, 2012[47] [ABSTRACT]	USA, 2006–11; Retrospective	ADA: 107; CZP: 28 (CD)	BMI<30–100 BMI≥30–35	Failure to respond to induction therapy, NR; 29.6%	Age: 38 (14) Sex: 37% Steroids: NR
Bultman, 2012[82]	The Netherlands; 2007–10; Prospective	ADA: 122 (CD)	BMI, median (range): 23 (15–42); Exposure categories: *per unit BMI*	Dose escalation; 38% required dose escalation; adjusted OR for need for dose escalation per unit increase in BMI: 1.11 [1.01–1.23] (variables adjusted for: sex, disease location, disease activity, perianal disease, prior anti-TNF, prior surgery, CRP)	Age: 35 (12) Sex: 44% Steroids: NR
Moore, 2011[62] [ABSTRACT]	USA, 2009–10; Retrospective	ADA: 19; CZP: 22 (CD)	BMI<25–21 (68.6%) BMI 25–29–20 (15.0%) BMI≥30–6 (16.4%)	Failure to achieve clinical remission or response based on HBI, median follow-up: 8 weeks); 19.5%	Age: 31 (13) Sex: 42% Steroids: Normal BMI– 20%; Obese or overweight– 38%
Qumseya, 2009[68] [ABSTRACT]	USA, NR; Retrospective	ADA: 118 (CD)	BMI<25–63 (53.4%) BMI 25–29–27 (22.9%) BMI≥30–28 (23.7%)	Failure of therapy (need for dose escalation or switching to alternative therapy), NR; 38.1%	Age: 24 (11) Sex: 36% Steroids: NR
**PSORIASIS and/or PSORIATIC ARTHRITIS**					
Zweegers, 2016[80]	The Netherlands, 2005–15; Prospective	ADA: 186; ETN: 238 (Psoriasis)	BMI, mean (SD): 27.8 (6.6); Exposure categories: BMI≥30 vs. BMI<30	Drug discontinuation due to ineffectiveness; 5-year discontinuation of ADA and ETN due to ineffectiveness, 46% and 55%, respectively (variables adjusted for: age, sex, trial eligible, family history for psoriasis, PsA, disease duration, baseline disease activity, prior therapy)	Age: ADA– 49 (13); ETN– 47 (13) Sex: ADA– 57%; ETN– 61% Steroids: NA
Villarasa, 2016[77]	Spain, 2007–13; Retrospective	ADA: 231; ETN: 248; IFX: 84 (Psoriasis)	BMI, mean (SD): 28.6 (5.9) BMI<30–334 (59.4%) BMI≥30–229 (40.6%)	Drug discontinuation due to primary non-response, loss of response or adverse events; 1-year drug discontinuation of ADA, ETN and IFX was 31%, 29.1% and 31.2%, respectively (variables adjusted for: drug use, baseline disease activity)	Age: 50 (13) Sex: 64% Steroids: NA
Ogdie, 2016[83] [ABSTRACT]	CORRONA Registry, USA, 2005–13; Prospective	Anti-TNF: 725	BMI, median (IQR): 30.9 (26.7–36.2); Exposure categories: *per unit BMI*	Failure to achieve remission (CDAI<2.8), 12m; 83%; each unit increase in BMI associated with 5% [2.0–8.4%] higher risk of failing to achieve remission (variables adjusted for: sex, prior therapy, baseline disease activity, work full time, pain)	Age, median (IQR): 52 (44–60) Sex: 44% Steroids: 14%
Menter, 2016[61]	PSOLAR, 2007–13; Prospective	ADA: 402; ETN: 289; IFX: 63 (Psoriasis)	BMI, mean (SD): 30.4 (7.1); Exposure categories: BMI≥35 vs. BMI 25–35 vs. BMI<25	Drug discontinuation, reported as time to event; NR	Age: 47 (14) Sex: 57% Steroids: NA
Hojgaard, 2016[26]	Denmark (DANBIO)/Iceland (ICEBIO), NR; Prospective	ADA: 520; ETN: 287; IFX: 352; CZP: 27; GLM: 85 (Psoriatic arthritis)	BMI<30–863 (68%) BMI≥30–408 (32%)	Failure to respond to therapy (EULAR goof response); discontinuation of therapy due lack of efficacy higher in obese vs. non-obese patients (HR, 1.85 [1.38–2.48])	Age: Obese– 49 (12); Non-obese– 47 (13) Sex: 45% Steroids: NR
Chiricozzi, 2016[46]	Italy, 2005–14; Retrospective	ADA: 316 (Psoriasis [n = 117] and psoriatic arthritis [n = 199])	BMI<25–123 (38.9%) BMI 25–29–138 (43.7%) BMI≥30–55 (17.4%)	Discontinuation of therapy; 46.8% discontinued therapy during the observation period	Age: 48 (13) Sex: 76% Steroids: NR
Warren, 2015[78]	BADBIR Registry, UK, 2007–14; Prospective	ADA: 1879 ETN: 1098 IFX: 96 (Psoriasis)	BMI<18.5–32 (0.9%) BMI 18.5–24.9–559 (16.1%) BMI 25–29–1024 (29.4%) BMI 30–34.9–788 (22.5%) BMI 35–40–464 (13.2%) BMI>40–346 (9.1%); Exposure categories: BMI≥30 vs. BMI 25–30 vs. BMI<25	Discontinuation of therapy due to ineffectiveness; 1-year drug discontinuation due to ineffectiveness rate, 13% (variables adjusted for age, sex, smoking status, comorbidities, disease duration, concomitant medications, biologic drug)	Age: 45 (13) Sex: 60% Steroids: NA
Di Lernia, 2014[50]	Italy; NR; Retrospective	Anti-TNF: 110 (Psoriasis)	BMI<25–123 (38.9%) BMI 25–29–138 (43.7%) BMI≥30–55 (17.4%)	Failure to respond to therapy (PASI50) by week 12–16; 13 patients (11.8%) did not respond	Age: 51 (12) Sex: NR Steroids: NA
Costa, 2014[49]	Italy, 2008–11; Prospective	Anti-TNF: 330 (Psoriatic arthritis)	BMI, mean (SD): Metabolic syndrome– 28.5 (4.5); No metabolic syndrome– 26.4 (3.8); Exposure categories: BMI≥30 vs. BMI<30	Failure to achieve minimal disease activity, 24 months; 52.2% failed to achieve minimal disease activity	Age: Metabolic syndrome– 47 (9); no metabolic syndrome– 47 (9) Sex: 45%; 46% Steroids: NR
Iannone, 2013[28]	Italy, 2006–11; Retrospective	ADA: 42; ETN: 48; IFX: 45 (Psoriatic arthritis)	BMI<25–43 (31.9%) BMI 25–29–47 (34.8%) BMI≥30–45 (33.3%)	Failure to achieve remission (DAS28<2.6), 36 months; 57.0% failed to achieve remission	BMI<25 vs. BMI 25–30 vs. BMI>30 Age: 51 (12) vs. 53 (11) vs. 56 (11) Sex: 47% vs. 57% vs. 47% Steroids: 83% vs. 50% vs. 38%
Di Minno, 2013[14]	Italy, 2007–10; Prospective	ADA: 80; ETN: 111; IFX: 79 (Psoriatic arthritis)	BMI<30–135 (50%) BMI 30–35–100 (37%) BMI>35–35 (13%)	Failure to achieve minimal disease activity, 12 months; 63.7% failed to achieve minimal disease activity (variables adjusted for: age, sex, disease duration, concomitant methotrexate, smoking, diabetes, hyperlipidemia, hypertension, ESR, CRP, baseline disease activity)	BMI<30 vs. BMI ≥30 Age: 51 (13) vs. 52 (10) Sex: 52% vs. 40% Steroids: NR
Di Renzo, 2012[51]	Italy, 2007–08; Prospective	ADA: 19; ETN: 28; IFX: 13 (Psoriasis [n = 36] and psoriatic arthritis [n = 24])	BMI<30–37 (46.3%) BMI≥30–43 (53.7%) [Only 60 patients completed study, and were reported]	Failure to achieve response (PASI75), 24 weeks; 23% failed to achieve response	Age: 39 (9) Sex: NR Steroids: NR
Naldi, 2008[63]	PSOCARE, Italy, 2005–07; Prospective	ETN: 810; IFX: 256	BMI<30 –ETN: 526 (64.9%); IFX: 186 (72.7%); BMI≥30 –ETN: 231 (28.5%); IFX: 43 (25.8%)	Failure to achieve response (PASI75), 8 weeks; 54.3% IFX-treated and 65.2% ETN-treated patients failed to achieve response	Age>40: 70.7% Sex: 66.6% Steroids: NA
Cassano, 2008[45]	APHRODITE, Italy, NR; Prospective	ADA: 144 (Psoriasis AND psoriatic arthritis)	BMI<25–57 (39.6%) BMI 25–30–55 (38.2%) BMI>30–32 (22.2%); Exposure categories: BMI≥30 vs. BMI<30	Failure to achieve response (PASI50), 12 weeks; 22.9% failed to respond	Age: 49 (NR) Sex: 51% Steroids: NR
**SPONDYLOARTHRITIS**					
Vidal, 2016[76] [ABSTRACT]	France, 2001–15; Retrospective	Anti-TNF: 168 (Axial SpA)	BMI–NR Exposure categories: BMI ≥25 vs. <25	Failure to achieve response (BASDAI50), 3 months; NR	Age: 42 (11) Sex: 24% Steroids: NR
Simone, 2014[74]	Italy, NR; Retrospective	Anti-TNF: 153 (Axial SpA)	BMI–NR Exposure categories: BMI ≥25 vs. <25	Failure to achieve strong clinical response (BASDAI<1), 12 months; NR	Age: 40 (8) Sex: 50% Steroids: NR
Gremese, 2014[31]	Italy, NR; Retrospective	ADA: 35; ETN: 31: IFX: 104 (Axial SpA)	BMI<25–92 (54.1%) BMI 25–30–55 (32.4%) BMI>30–23 (13.5%)	Failure to achieve response (BASDAI50), 12 months; 38.8%	Age: 40 (12) Sex: 69% Steroids: NR
Ottaviani, 2012[64]	France, NR; Retrospective	IFX: 155	BMI<25–63 (40.6%) BMI 25–30–54 (34.8%) BMI>30–38 (24.6%)	Failure to achieve response (BASDAI50), 6 months; 44.6%	Age, median (IQR): 43 (35–52) Sex: 63% Steroids: NR

ADA = Adalimumab; ASAS = Assessment of Spondyloarthritis International Society; BASDAI = Bath Ankylosing Spondylitis Disease Activity Index; BMI = Body mass index; CAI = Clinical activity index; CDAI = Crohn’s disease activity index; CRP = C-reactive protein; CS = corticosteroids; CZP = Certolizumab pegol; ESR = Erythrocyte sedimentation rate; ETN = Etanercept; EULAR = European League Against Rheumatism; hfPGA = hand/feet physician global assessment; IFX = Infliximab; IM = Immunomodulators; MTX = Methotrexate; NR = Not reported; PASI = Psoriasis Area Severity Index; qw = every week; qow = every other week; SD = standard deviation; SpA = Spondyloarthropathy

### Obesity and response to anti-TNF therapy

On meta-analysis, across included IMIDs and across all anti-TNF agents, obesity was associated with 60% higher odds of failure of index anti-TNF therapy (OR, 1.60; 95% CI, 1.39–1.83), with substantial heterogeneity (I^2^ = 71%).[14, 26–33, 40–80]

#### Dose-response relationship

**Fig 2:**
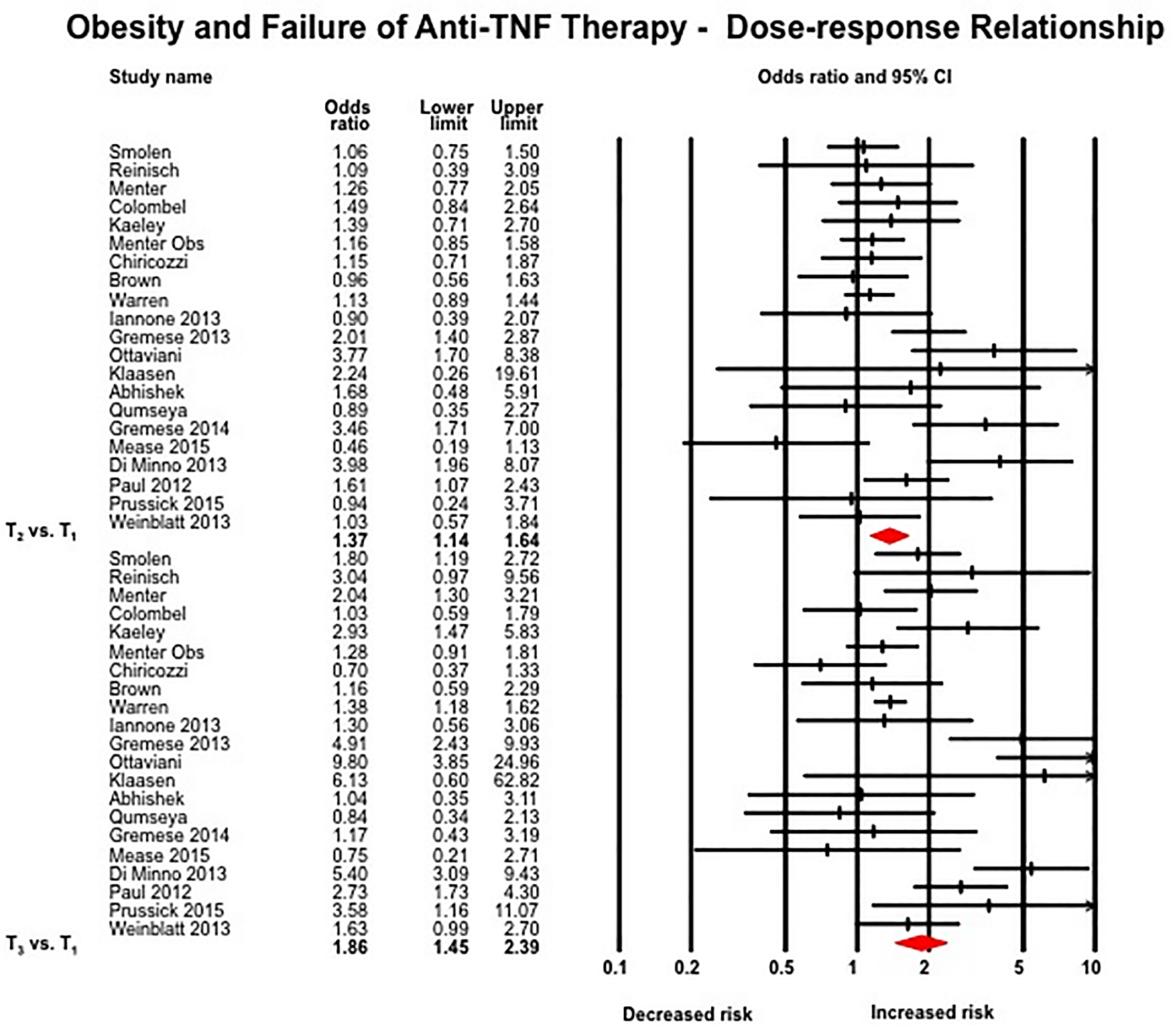
Association between obesity and response to anti-TNF therapy–dose-response relation, with comparison of patients in the 3^rd^ tertile by weight or BMI with patients in the 1^st^ tertile, and of patients in the 2^nd^ tertile vs. the first tertile.

The odds of failing therapy were highest in the third tertile of BMI or weight as compared to patients in the second tertile (21 studies; T_3_ vs. T_1_: OR, 1.86 [1.45–2.39]; T_2_ vs. T_1_: OR, 1.37 [1.14–1.65], p-value comparing effect size for T_3_ and T_2_ = 0.05) (Fig 2).[14, 28–32, 40, 43, 46, 48, 57, 59–61, 65, 67–69, 75, 78, 79] On restricting analyses only to studies that reported dose-response in terms of WHO-defined categories (n = 17 studies), similar results were observed (obese vs. normal BMI: OR, 1.87 [1.39–2.52]; overweight vs. normal BMI: OR, 1.38 [1.11–1.73], p-value comparing effect size for obese and overweight = 0.11).[14, 28–32, 40, 43, 46, 57, 59–61, 67, 68, 75, 78] On analysis of studies reporting effect estimates per unit BMI, each 1 kg/m^2^ increase in BMI was associated with 6.5% higher odds of failing anti-TNF therapy (9 studies; OR, 1.065 [1.043–1.087], I^2^ = 5%) (S1 Fig).[32, 33, 42, 54, 81–84]

#### Subgroup analyses

**Fig 3:**
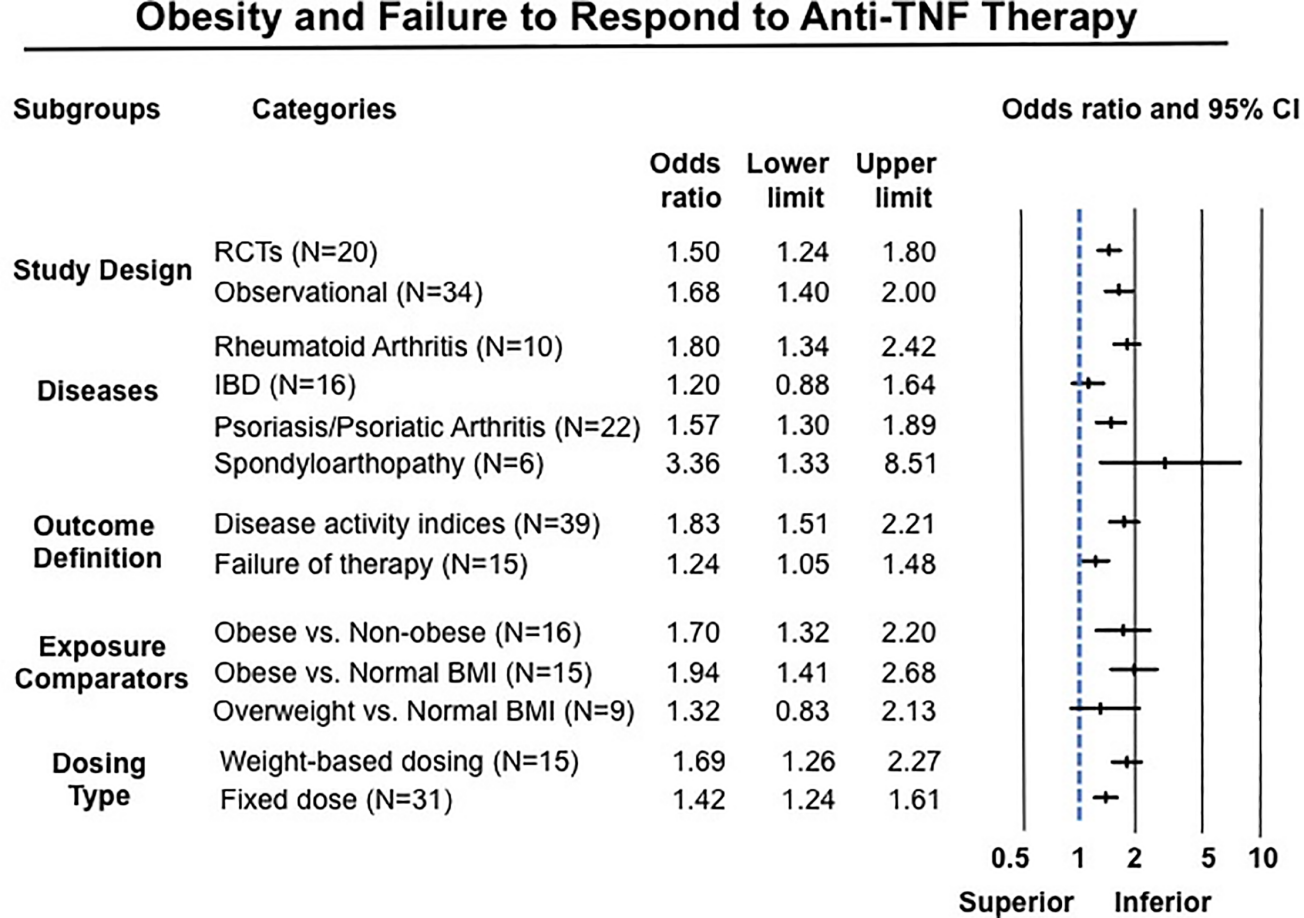
Summary results of subgroups analyses based on study design, disease type, outcome definition, exposure categories and drug dosing.

Results of subgroup analyses are summarized in Fig 3.

#### Types of diseases

**Fig 4:**
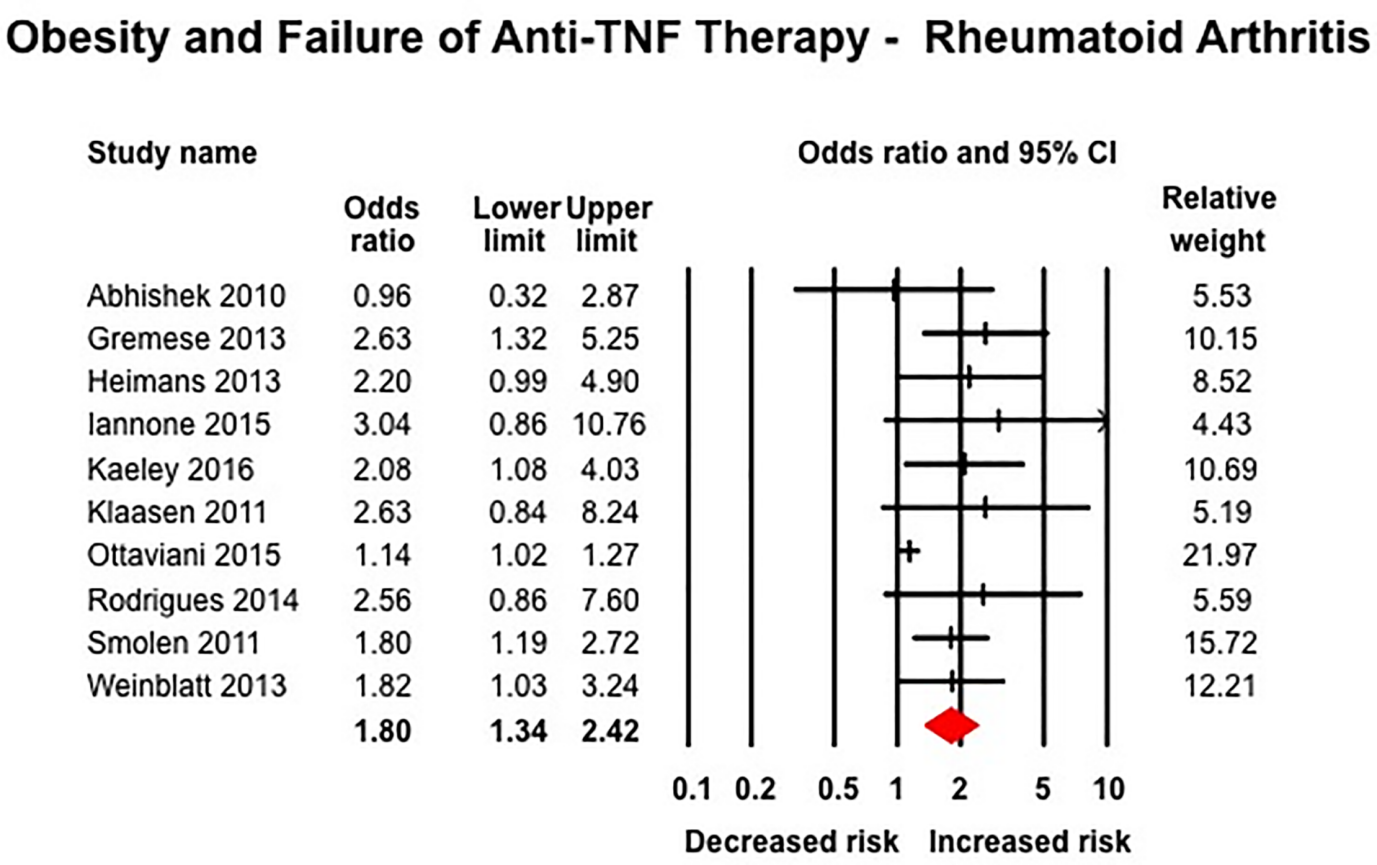
Obesity and response to anti-TNF therapy in patients with rheumatoid arthritis.

**Fig 5:**
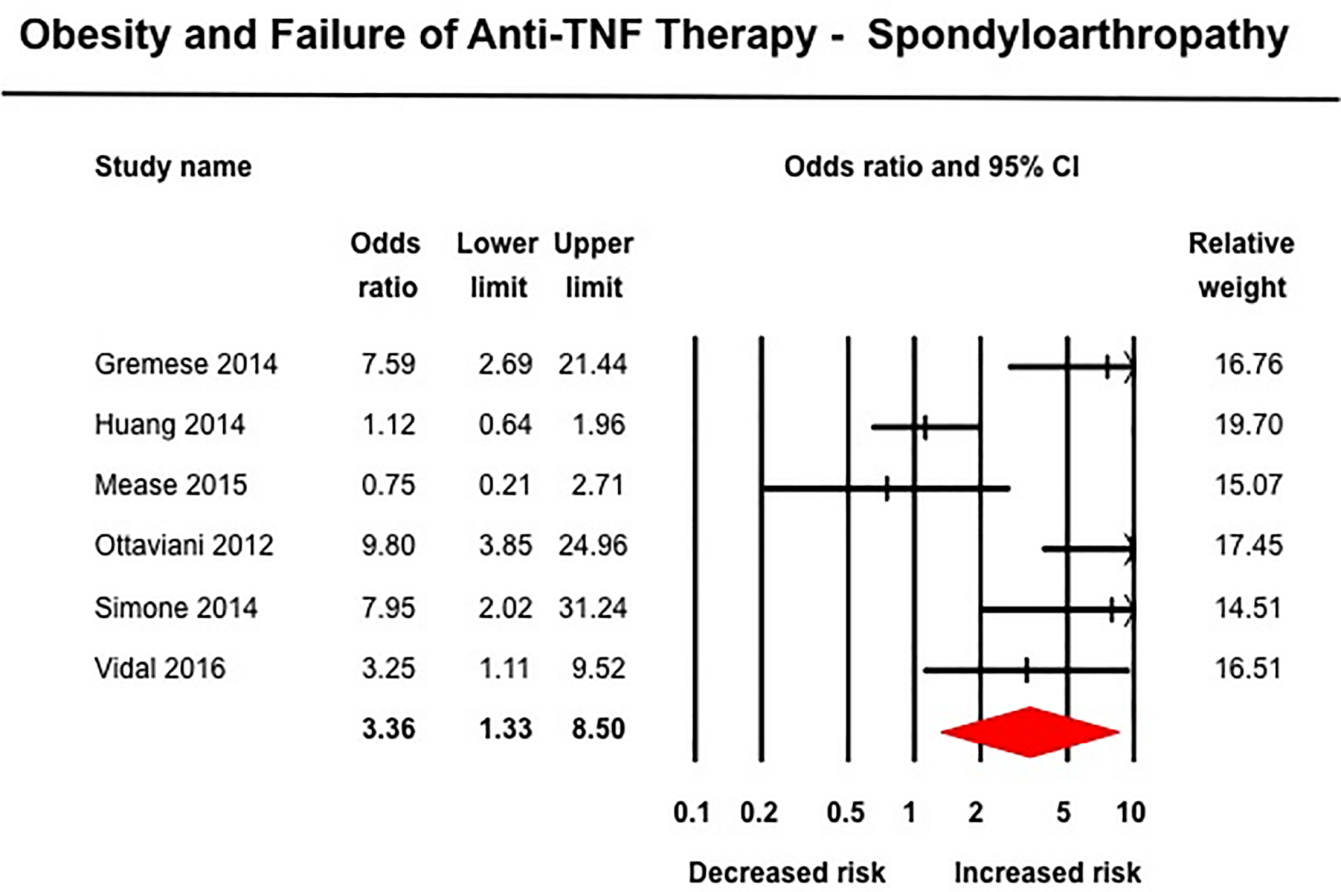
Obesity and response to anti-TNF therapy in patients with spondyloarthritis.

**Fig 6:**
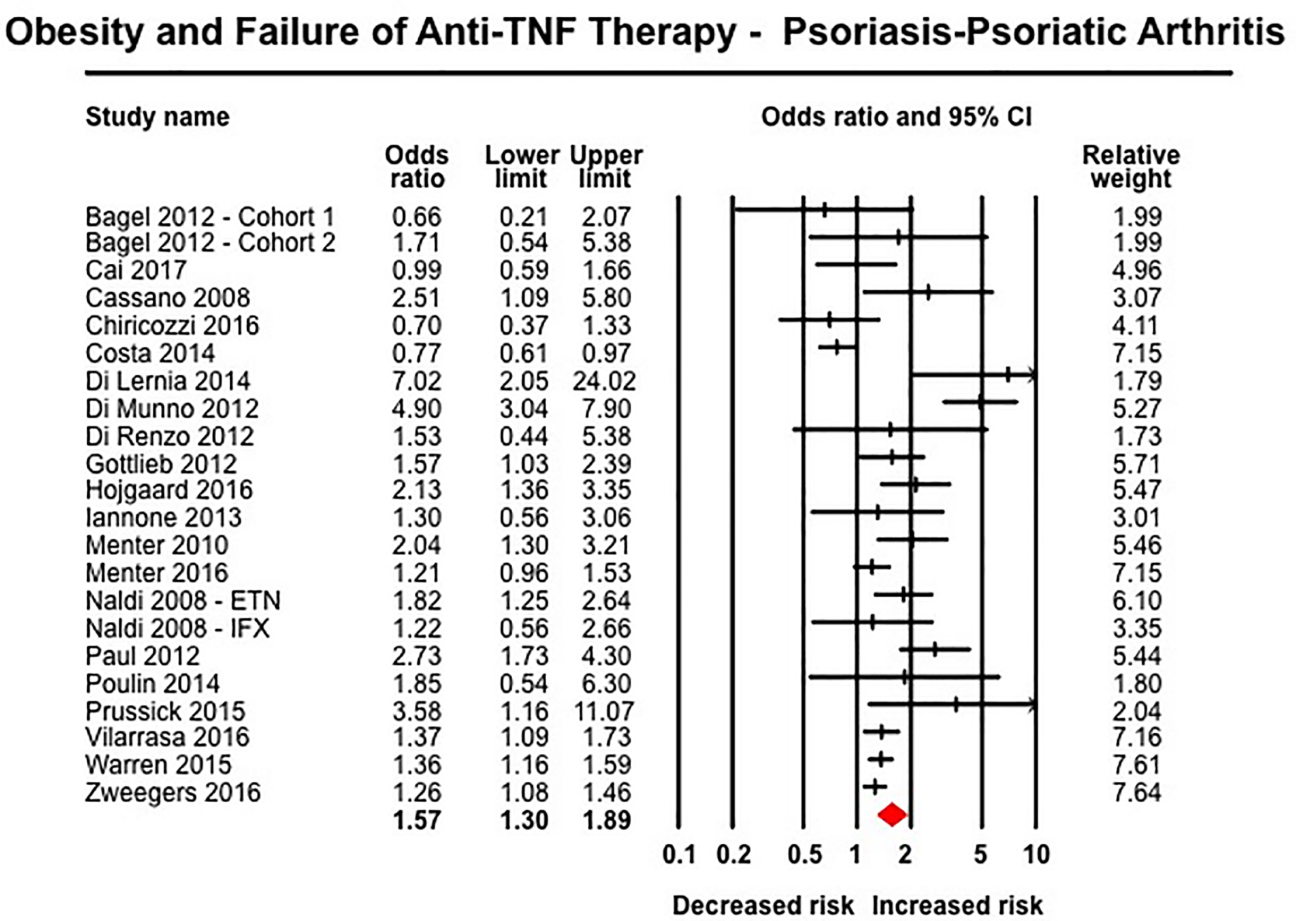
Obesity and response to anti-TNF therapy in patients with psoriasis and psoriatic arthritis.

**Fig 7:**
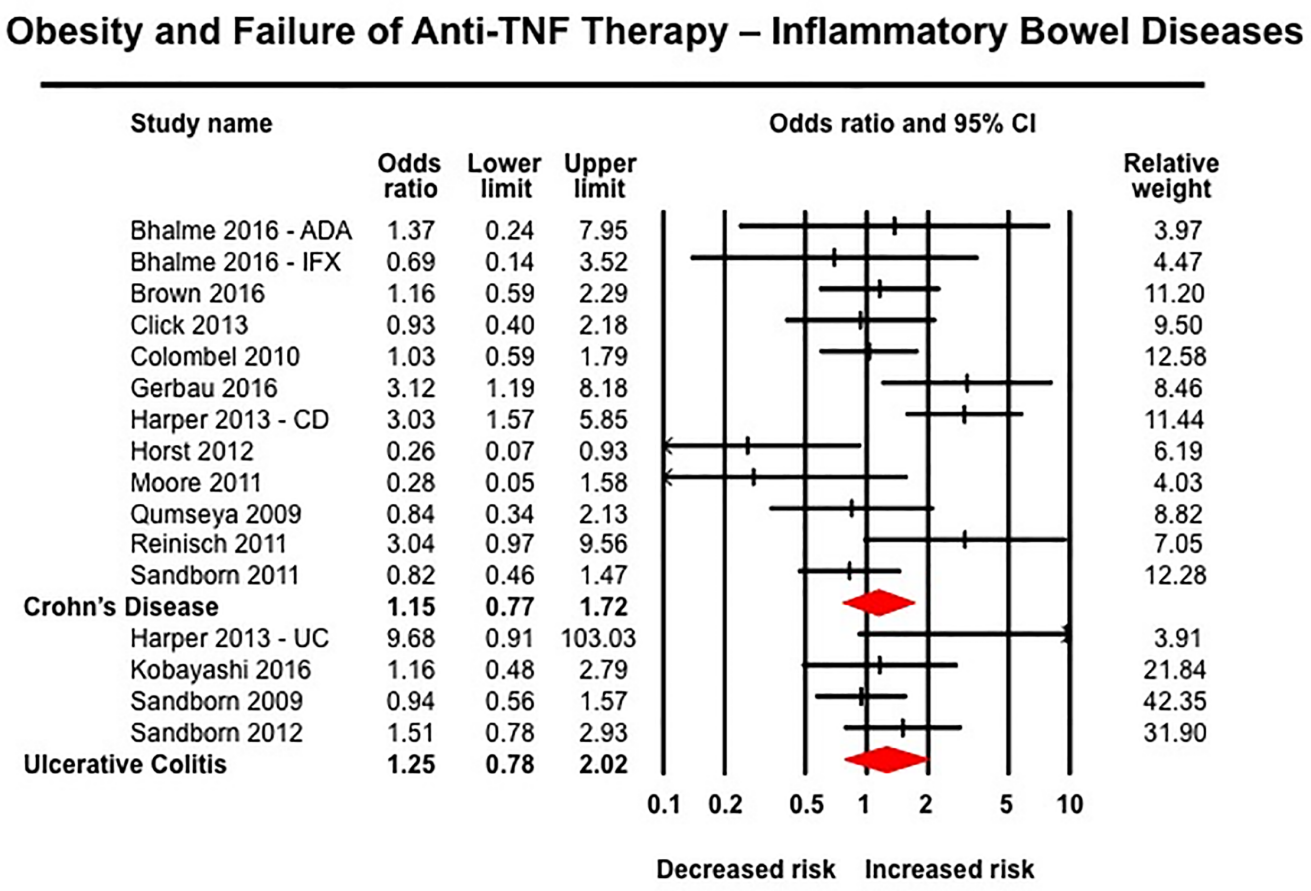
Obesity and response to anti-TNF therapy in patients with inflammatory bowel diseases.

Obesity was associated with inferior response to anti-TNF therapy across all rheumatic diseases. In 10 studies in patients with RA including 3,403 patients (median [IQR], 23% [17–29%] obese), obese patients had 80% higher odds of failure of therapy (OR, 1.80 [1.34–2.42]; I^2^ = 58%) (Fig 4).[27, 29, 30, 32, 40, 54, 57, 70, 75, 79] Similarly, obese patients with SpA (6 studies, 966 patients, 14% obese) (OR, 3.36 [1.33–8.51], I^2^ = 81%) (Fig 5)[31, 56, 59, 64, 74, 76] and psoriasis-PsA (22 studies, 11,873 patients, median [IQR], 33% [22–50%]) (OR, 1.57 [1.30–1.89], I^2^ = 77%) had inferior response to anti-TNF therapy (Fig 6).[14, 26, 28, 41, 44–46, 49–52, 60, 61, 63, 65–67, 77, 78, 80] No significant association was observed between obesity and response to anti-TNF therapy in patients with IBD based on 16 studies, with 3,130 patients (median [IQR], 16.5% [16–24%] obese) (OR, 1.20 [0.88–1.64], I^2^ = 50%) (Fig 7), either in patients with CD (12 studies; OR, 1.15 [0.77–1.72]) or ulcerative colitis (4 studies; OR, 1.25 [0.78–2.02]).[33, 42, 43, 47, 48, 53, 55, 58, 62, 68, 69, 71–73] Of note, amongst IBD patients, 8/16 studies reported only dichotomous exposure assessment, generally categorized as above or below median weight and as overweight vs. non-overweight categories; more extreme weight categories were not reported.

#### Route of administration and dosing regimen

Infliximab is the only anti-TNF agent routinely administered intravenously in a weight-based dose; additionally, a form of golimumab (Simponi Aria^®^) is occasionally administered intravenously in a weight-based dose; all other agents are administered in a fixed dose independent of body weight, subcutaneously. Hence, route and dosing scheme could not be evaluated independently. Patients with obesity treated with both weight-based dosing regimens (16 studies; OR, 1.69 [1.26–2.27], I^2^ = 67%) (Panel A in S2 Fig)[26, 29, 30, 33, 42, 43, 48, 53, 54, 58, 63, 64, 71–73, 79] and fixed-dose regimens (31 studies; OR, 1.42 [1.24–1.61], I^2^ = 46%) (Panel B in S2 Fig)[26, 27, 32, 41, 42, 44–47, 52, 53, 55–57, 59–63, 65–69, 72, 73, 75–78, 80] had higher odds of failing therapy, without significant differences in the summary estimate (p-value comparing effect size for fixed-dose and weight-based dosing = 0.28).

#### Exposure categories and outcome definition

Studies comparing patients with obesity with either non-obese (16 studies, OR, 1.70 [1.32–2.20])[26, 29, 31–33, 42, 45, 47, 49–51, 63, 70, 77] or normal BMI (15 studies, OR, 1.94 [1.41–2.68])[14, 27, 28, 30, 43, 46, 53, 57, 59, 60, 64, 67, 68, 75, 78] patients consistently reported a negative effect of obesity on response to therapy; in contrast studies with exposure categories of only overweight vs. non-overweight (without more extreme BMI categories) did not observe a significant association (9 studies; OR, 1.32 [0.82–2.13]) (p_interaction_ = 0.42).[33, 44, 54, 55, 61, 62, 72, 74, 76] Based on outcome definition, the effect of obesity was more pronounced in studies that used validated definitions of clinical remission or response (based on disease activity indices; 39 studies) (failure to achieve remission/response: OR, 1.83 [1.51–2.21])[14, 26–32, 40, 41, 44, 45, 47–52, 54, 56–60, 63–67, 69, 70, 72–76, 79] as compared to studies that used pragmatic definition of treatment modification (15 studies; OR, 1.24 [1.05–1.48]) (p_interaction_<0.01).[33, 42, 43, 46, 53, 55, 61, 62, 68, 71, 77, 78, 80]

#### Study design

Obesity was associated with inferior response to anti-TNF therapy in both RCTs (20 studies; OR, 1.50 [1.24–1.80], I^2^ = 40%) (Panel A in S3 Fig)[41, 44, 48, 52, 54, 56–60, 65–67, 69, 71–73, 75, 79] and observational studies (34 studies; OR, 1.68 [1.48–2.00], I^2^ = 78%) (Panel B in S3 Fig), without significant difference between subgroups (p_interaction_ = 0.39).[14, 26–33, 40, 42, 43, 45–47, 49–51, 53, 55, 61–64, 68, 70, 74, 76–78, 80]

On meta-regression, prevalence of obesity in included studies modestly but significantly affected summary estimate (p = 0.06), with smaller effect size observed in studies with lower prevalence of obesity (Panel A in S4 Fig); prevalence of outcome did not affect summary estimate (p = 0.54) (Panel B in S4 Fig).

### Sensitivity analysis and publication bias

Overall results were similar when restricting to studies that reported analyses adjusted for key confounding variables (13 studies; OR, 1.52 [1.22–1.89], I^2^ = 79%),[14, 29, 32, 33, 40, 43, 55, 70, 71, 77, 78, 80] studies focusing only on failure to achieve remission based on standard DAI (22 studies; OR, 1.62 [1.26–2.08], I^2^ = 73%)[14, 27, 28, 32, 41, 44, 48, 49, 52, 57, 58, 60, 63, 66, 69, 70, 72, 73, 75, 76] and on limiting to studies published as full-texts (45 cohorts; OR, 1.61 [1.40–1.86], I^2^ = 73%).[14, 26–33, 40–46, 48–52, 54, 56, 58–61, 63–67, 69, 71–74, 77–80] There was no evidence of publication bias based on examination of funnel plot symmetry (S5 Fig) and quantitatively on Begg and Mazumdar’s test (p = 0.60).

## Discussion

### Principal findings

In this systematic review with meta-analysis of 54 cohorts with 19,372 patients with select IMIDs treated with anti-TNF agents, we made several key observations related to the influence of obesity on response to anti-TNF therapy. First, we observed that obesity was associated with a 60% higher odds of failing anti-TNF therapy as compared to non-obese and normal BMI counterparts, for most IMIDs, including RA, spondyloarthropathies and psoriasis and psoriatic arthritis, but not IBD. This association was stable across diverse subgroups including study design, exposure definition, outcome measurement, and was independent of other potential confounding variables. Second, a dose-dependent effect was seen, with each unit increase in BMI associated with 6.5% higher odds of failing therapy. Third, this effect was independent route of administration and dosing scheme. It is also important to note that <10% of eligible RCTs reported results stratified by baseline BMI. Our findings of negative impact of obesity on treatment response to anti-TNF agents suggests that obesity is a negative prognostic factor and treatment effect modifier that must be considered in clinical trial design and clinical practice.

Obesity is recognized as a perpetual state of chronic low-grade inflammation, through systemic and paracrine increase in levels of cytokines, chemokines and adipokines.[6] Besides its direct impact on inflammation, obesity can also modify pharmacokinetics of anti-TNF and other biologic agents. Population pharmacokinetic studies of all anti-TNF agents have identified high body weight as a risk factor associated with increased clearance of drug, resulting in shorter half-life and lower serum trough drug concentrations.[20–22] This effect might be related to rapid proteolysis and to a ‘TNF-sink’ phenomenon, wherein the clearance of a monoclonal antibody that binds to membrane antigen is faster at low doses as the unbound targets “sop up” antibody, serving as a sink.[85] This may explain why patients with obesity treated even weight-based regimens such as infliximab, had inferior response to therapy.

### Comparison with other studies

Our findings expand on prior observations on the potentially negative impact of obesity on overall treatment response in patients with RA and psoriasis. However, these prior studies included all treatment categories, instead of focusing on anti-TNF therapy. Moreover, they were unable to simultaneously study differential impact across different diseases, and with different anti-TNF agent. Though we had hypothesized that obesity would negatively impact response to anti-TNF therapy across all selected diseases, we did not observe such an association in patients with IBD. This may be a true observation. Patients with IBD show a unique locally restrictive form of visceral adipose tissue—creeping fat—whereby mesenteric fat hyperplasia is limited to areas of inflamed bowel.[86, 87] It is likely that in IBD patients, this local mesenteric fat plays a more important role than systemic obesity. Additionally, dose of infliximab and adalimumab approved in patients with IBD is higher than that for other rheumatic diseases. Differential effect of confounding by disease severity in IBD and rheumatic diseases may also explain this finding–severe IBD is likely to result in weight loss and misclassification of obesity, whereas, severe rheumatic diseases would likely impact physical activity, promote sedentary lifestyle and contribute to obesity. Alternatively, it is possible that our observation in IBD patients is biased. Most studies in IBD patients presented dichotomous categories, usually as above or below median weight, and did not include patients at extreme categories of BMI, where the negative impact of obesity may be more pronounced. On meta-regression, effect estimates were smaller in studies with lower prevalence of obesity. Five studies in IBD patients were reported only as abstracts, putting them at high risk of bias (though excluding these studies did not modify the summary estimate). Further studies, of both fixed dose therapies and weight-based therapies, preferably using individual participant data from clinical trials are warranted to better understand this association.

### Strengths and limitations

The strengths of this systematic review include: (a) comprehensive and systematic literature search with well-defined inclusion criteria, including both RCTs and observational studies as cohort studies, (b) *a priori* comprehensive sub-group and sensitivity analyses to evaluate the stability of findings and identify potential factors responsible for inconsistencies, (c) assessment of dose-response relationship using two approaches, and (d) simultaneous evaluation of unadjusted (based on raw numbers) and adjusted risk estimates, and hence, being able to evaluate the potential influence of measured confounders on the summary estimate.

There are several limitations in our study. First, the meta-analysis included only cohorts, derived from both RCTs and observational studies, but participants in RCTs were not stratified based on presence or absence of obesity. Most of the included studies did not account for several potential confounding factors including steroid use and baseline disease activity, both of which influence exposure and outcome. Additionally, factors that may be intrinsically related to obesity, such as depression, fibromyalgia, etc., may confound this association between obesity and inferior clinical response to therapy. While sensitivity analysis using the adjusted data had little effect on the summary OR, potentially suggesting that any difference attributable to using different confounders for adjustment is likely small, data regarding several confounders was inadequately reported. Second, substantial heterogeneity was observed in the overall analysis, which was partly explained by difference in outcome definition and prevalence of obesity in included cohorts, as well as different types of diseases. It is important to note that a stronger effect estimate was observed prospective studies that used validated disease activity indices, with fairly consistent outcome definitions, as compared to retrospective studies which used non-validated definitions such as need for escalation of index therapy, switching to alternative therapy or surgery, etc. Fourth, despite absence of small study effects, there was concern for reporting bias. Less than 10% of eligible RCTs results stratified by baseline BMI; when they did, exposure was frequently classified as dichotomous variable, above and below a median weight. Finally, we limited our analysis to only anti-TNF agents, instead of including other non-TNF-biologic agents and small molecules. This was done to minimize conceptual heterogeneity. Negative impact of obesity on response to other therapies has been observed, and merits detailed evaluation.

### Implications for clinical practice

While the negative impact of obesity in patients with psoriasis is well-known, our observations that obesity uniformly results in inferior response to anti-TNF therapy across all rheumatic diseases that we studied has important implications for both clinical practice and clinical trial design. In clinical practice, physicians may consider aggressive treatment and close proactive monitoring in patients with obesity treated with anti-TNF agents such as empirically using higher dose of anti-TNF agent in obese patients, frequent therapeutic drug monitoring and/or use of combination therapy with immunomodulators to increase drug concentration and decrease risk of immunogenicity. Clinical trialists should consider obesity as a potential effect modifier and consider obesity as a stratification variable; at the very least, stratified analysis by baseline BMI should be performed and consistently reported. Finally, the presence of obesity may offer a potential therapeutic intervention, either through adjusting biologic dosing for body weight or through directly targeting the obesity itself for treatment in patients with select IMIDs with a multi-disciplinary approach. Small RCTs and cohort studies in patients with psoriasis, psoriatic arthritis and RA have suggested a beneficial effect of intentional weight loss on treatment response to anti-TNF agents.[88–90]

### Conclusion

In conclusion, obesity is associated with inferior response to anti-TNF therapy in patients with rheumatic diseases, but not in patients with IBD. This effect is independent of route of administration, and is observed in patients treated with weight-based as well as fixed dose agents. However, current findings need to be interpreted with caution due to high heterogeneity and due to lack of adjustment for relevant confounding factors in included studies. Prospective cohort studies and post-hoc analyses of RCTs with individual participant level data are warranted to confirm this association. If this effect is consistent, interventional studies targeting obesity should be explored for difficult-to-treat obese patients with select IMIDs.

## Supporting information

S1 Text PRISMA checklist. (DOCX)

S2 Text Search strategy for observational studies. (DOCX)

S3 Text Search strategy for randomized controlled trials. (DOCX)

S4 Text Data abstraction. (DOCX)

S1 Table Risk of bias assessment. (DOCX)

S1 Fig Association between obesity measured as per unit increase in body mass index and response to anti-TNF therapy. (TIFF)

S2 Fig Association between obesity and response to anti-TNF therapy–Subgroup analyses based on anti-TNF dosing regimen: (A) weight-based dosing, and (B) fixed dose therapies.
(TIFF)

S3 Fig Association between obesity and response to anti-TNF therapy–Subgroup analyses based on study design: (A) RCTs, and (B) observational studies.
(TIFF)

S4 Fig Meta-regression based on (A) prevalence of obesity, and (B) prevalence of outcome.
(TIFF)

S5 Fig Funnel plot to evaluate publication bias. (TIFF)
