# Correction: Obesity and response to anti-tumor necrosis factor-α agents in patients with select immune-mediated inflammatory diseases: A systematic review and meta-analysis

**DOI:** 10.1371/journal.pone.0203499

**Published:** 2018-08-29

**Authors:** The PLOS ONE Staff

There is an error in the XML that is causing the third author’s name to be indexed incorrectly. The publisher apologizes for this error. The name should be indexed as: Vande Casteele, N. The correct citation is: Singh S, Facciorusso A, Singh AG, Vande Casteele N, Zarrinpar A, Prokop LJ, et al. (2018) Obesity and response to anti-tumor necrosis factor-α agents in patients with select immune-mediated inflammatory diseases: A systematic review and meta-analysis. PLoS ONE 13(5): e0195123. https://doi.org/10.1371/journal.pone.0195123
