# Supplementary material for: Obesity and response to anti-tumor necrosis factor-α agents in patients with select immune-mediated inflammatory diseases: A systematic review and meta-analysis
Source: PLoS One. 2018 May 17;13(5):e0195123. doi: 10.1371/journal.pone.0195123 (PMC5957395; doi:10.1371/journal.pone.0195123)
Supplement: S2 Text — (DOCX) [file pone.0195123.s002.docx]

**S2 Text. Actual Search Strategy – Observational Studies**

Ovid

Database(s): Embase 1988 to 2017 Week 03, EBM Reviews - Cochrane Database of Systematic Reviews 2005 to January 18, 2017, Epub Ahead of Print, In-Process & Other Non-Indexed Citations, Ovid MEDLINE(R) Daily and Ovid MEDLINE(R) 1946 to Present
Search Strategy:

| **#** | **Searches** | **Results** |
| --- | --- | --- |
| 1 | Autoimmune Diseases/ | 76681 |
| 2 | ("auto immune disease*" or "auto immunologic disease*" or "autoaggressive disease*" or "autoantibody disease*" or "autoimmune disease*" or "autoimmune disorder*" or "auto-immune disorder*" or "autoimmune disturbance*" or "autoimmune pathology" or "autoimmuno disease*" or "autoimmunologic disease*").mp. | 214953 |
| 3 | exp Arthritis, Rheumatoid/ | 262848 |
| 4 | ("arthritis deformans" or "arthrosis deformans" or "beauvais disease" or "chronic articular rheumatism" or "chronic polyarthritis" or "chronic progressive poly arthritis" or "chronic progressive polyarthritis" or "Felty syndrome" or "Feltys syndrome" or "fereol node" or "inflammatory arthritis" or "meynet node" or rheumarthritis or "rheumatic arthritis" or "rheumatic fever nodule" or "rheumatic nodule" or "rheumatic polyarthritis" or "rheumatic skin disorder" or "rheumatoid arthritis" or "rheumatoid nodule" or "Still disease" or "Stills disease").mp. | 270785 |
| 5 | exp Inflammatory Bowel Diseases/ | 180705 |
| 6 | enteritis/ | 60621 |
| 7 | exp Crohn disease/ | 105448 |
| 8 | exp ulcerative colitis/ | 85819 |
| 9 | ("inflammatory bowel disease*" or "ulcerative colitis" or "crohn disease*" or "crohns disease*" or enteritis or "inflammatory enteropath*" or "cleron disease" or "morbus crohn" or "regional enterocolitis" or "colitis ulcerativa" or "colitis ulcerosa" or "mucosal colitis" or "ulcerous colitis" or "ulcerative colorectitis" or "ulcerative procto colitis" or "ulcerative proctocolitis").tw. | 209137 |
| 10 | exp Psoriasis/ | 87184 |
| 11 | ("Andrews disease" or "arthritic psoriases" or "arthritic psoriasis" or "palmar plantar bacterid" or "palmoplantar psoriases" or "palmoplantar psoriasis" or "palmoplantar pustuloses" or "palmoplantar pustulosis" or "palmoplantaris pustuloses" or "palmoplantaris pustulosis" or parapsoriases or parapsoriasis or "peripheral pustuloses" or "peripheral pustulosis" or "plantar palmar bacterid" or psoriases or "psoriasiform dermatitis" or "psoriasiform dermatoses" or "psoriasiform dermatosis" or "psoriasiform lesion*" or "psoriasiform rash" or "psoriasiform skin rash" or psoriasis or "psoriasis arthropathica" or "psoriatic arthritis" or "psoriatic arthropath*" or "psoriatic epidermis" or "psoriatic skin" or "pustular bacterid" or "pustulosis of palms and soles" or "pustulosis palmaris" or "pustulosis palmaris et plantaris" or "pustulosis palmoplantaris" or "pustulosis plantaris" or "recalcitrant pustular eruption*" or "willan lepra").mp. | 110710 |
| 12 | 10 or 11 | 111418 |
| 13 | exp Arthritis, Psoriatic/ | 21156 |
| 14 | ("alibert bazin disease" or "arthritic psoriasis" or "arthritis psoriatica" or "arthropathic psoriasis" or "psoriasis arthropathica" or "psoriasis pustulosa arthropathica" or "psoriatic arthritis" or "psoriatic arthropath*" or "psoriatic polyarthritis" or "psoriatic rheumatism" or "psoriatic rheumatoid arthritis").mp. | 24411 |
| 15 | exp Spondylarthropathies/ | 29143 |
| 16 | ("bechterew syndrome" or "marie strumpell spondylitis" or "marie-strumpell spondylitis" or spondylarthropath* or spondyloarthropath*).mp. | 10470 |
| 17 | exp Spondylitis, Ankylosing/ | 34774 |
| 18 | ("ankylating spondylitis" or "ankylopoietic spondylarthritis" or "ankylopoietic spondylitis" or "ankylosing spine" or "ankylosing spondilitis" or "Ankylosing Spondylarthritides" or "Ankylosing Spondylarthritis" or "ankylosing spondylarthrosis" or "Ankylosing Spondylitis" or "Ankylosing Spondyloarthritides" or "Ankylosing Spondyloarthritis" or "ankylosis spondylitis" or "ankylotic spondylitis" or "Bechterew Disease" or "Bechterews Disease" or "bekhterev disease" or "bekhterevs disease" or "Marie-Struempell Disease" or "Marie-Struempells Disease" or "morbus bechterew" or "Rheumatoid Spondylitis" or "spinal ankylosis" or "spine ankylosis" or "Spondylarthritis Ankylopoietica" or "spondylarthritis ankylosans" or "spondylarthrosis ankylopoietica" or "spondylitis ankylopoetica" or "Spondylitis Ankylopoietica" or "Spondyloarthritis Ankylopoietica" or "vertebral ankylosis").mp. | 36138 |
| 19 | or/1-18 | 852264 |
| 20 | exp infliximab/ | 48901 |
| 21 | exp adalimumab/ | 27711 |
| 22 | exp certolizumab pegol/ | 4912 |
| 23 | exp golimumab/ | 4204 |
| 24 | exp etanercept/ | 30845 |
| 25 | exp abatacept/ | 9441 |
| 26 | exp tocilizumab/ | 6618 |
| 27 | exp rituximab/ | 70403 |
| 28 | exp tofacitinib/ | 1781 |
| 29 | exp ustekinumab/ | 3963 |
| 30 | exp secukinumab/ | 1153 |
| 31 | exp ixekizumab/ | 560 |
| 32 | exp brodalumab/ | 507 |
| 33 | exp alefacept/ | 1703 |
| 34 | (abatacept or adalimumab or alefacept or antegren or antiTNF or "anti-TNF" or "antitumor necrosis factor" or "anti-tumor necrosis factor" or "antitumour necrosis factor" or "anti-tumour necrosis factor" or avakine or biologic or biologics or brodalumab or "certolizumab pegol" or cimzia or "cnto 1275" or cnto1275 or etanercept or golimumab or humira or inflectra or infliximab or ixekizumab or "ldp 02" or ldp02 or "mln 0002" or "mln 02" or mln0002 or mln02 or "monoclonal antibody D2E7" or "pegylated tumor necrosis factor" or remicade or remsima or revellex or rituximab or secukinumab or simponi or stelara or tocilizumab or tofacitinib or trudexa or tysabri or ustekinumab).mp. | 303652 |
| 35 | or/20-34 | 303652 |
| 36 | 19 and 35 | 97005 |
| 37 | exp Obesity/ | 599699 |
| 38 | exp Weight Loss/ | 184673 |
| 39 | exp body mass index/ | 413139 |
| 40 | (adipos* or antiobesity or "anti-obesity" or BMI or "body ban mass" or "body mass ind*" or "body mass index" or "body weight" or bodyweight or obes* or obesitas or "over eat*" or "over fed" or "over feed*" or "over weight" or overeat* or overfed or overfeed* or "overload syndrom*" or overweight or "quetelet index" or "quetelets index" or "skinfold thickness" or weight or "weight cycling").mp. | 2973463 |
| 41 | 37 or 38 or 39 or 40 | 3044931 |
| 42 | 19 and 35 and 41 | 6724 |
| 43 | exp meta analysis/ | 243708 |
| 44 | exp Meta-Analysis as Topic/ | 55179 |
| 45 | exp "systematic review"/ | 151670 |
| 46 | ((meta adj analys*) or (systematic* adj3 review*)).mp,pt. | 535944 |
| 47 | exp Cohort Studies/ | 2123844 |
| 48 | exp prospective study/ | 884431 |
| 49 | longitudinal study/ | 232981 |
| 50 | exp retrospective study/ | 1196355 |
| 51 | (cohort* or "longitudinal study" or "longitudinal survey" or "longitudinal analysis" or "longitudinal evaluation" or ((retrospective or "ex post facto") adj3 (study or survey or analysis or design)) or "prospective study" or "prospective survey" or "prospective analysis" or (("follow-up" or followup) adj (stud* or survey or analysis))).mp,pt. | 3179188 |
| 52 | or/43-51 | 4291756 |
| 53 | 42 and 52 | 1831 |
| 54 | limit 53 to ("all adult (19 plus years)" or "young adult (19 to 24 years)" or "adult (19 to 44 years)" or "young adult and adult (19-24 and 19-44)" or "middle age (45 to 64 years)" or "middle aged (45 plus years)" or "all aged (65 and over)" or "aged (80 and over)") [Limit not valid in Embase,CDSR; records were retained] | 1735 |
| 55 | limit 54 to (adult <18 to 64 years> or aged <65+ years>) [Limit not valid in CDSR,Ovid MEDLINE(R),Ovid MEDLINE(R) Daily Update,Ovid MEDLINE(R) In-Process,Ovid MEDLINE(R) Publisher; records were retained] | 871 |
| 56 | limit 53 to ("all infant (birth to 23 months)" or "all child (0 to 18 years)" or "newborn infant (birth to 1 month)" or "infant (1 to 23 months)" or "preschool child (2 to 5 years)" or "child (6 to 12 years)" or "adolescent (13 to 18 years)") [Limit not valid in Embase,CDSR; records were retained] | 1564 |
| 57 | limit 56 to (embryo or infant or child or preschool child <1 to 6 years> or school child <7 to 12 years> or adolescent <13 to 17 years>) [Limit not valid in CDSR,Ovid MEDLINE(R),Ovid MEDLINE(R) Daily Update,Ovid MEDLINE(R) In-Process,Ovid MEDLINE(R) Publisher; records were retained] | 337 |
| 58 | 57 not 55 | 146 |
| 59 | 53 not 58 | 1685 |
| 60 | (exp animals/ or exp nonhuman/) not exp humans/ | 9069979 |
| 61 | ((alpaca or alpacas or amphibian or amphibians or animal or animals or antelope or armadillo or armadillos or avian or baboon or baboons or beagle or beagles or bee or bees or bird or birds or bison or bovine or buffalo or buffaloes or buffalos or "c elegans" or "Caenorhabditis elegans" or camel or camels or canine or canines or carp or cats or cattle or chick or chicken or chickens or chicks or chimp or chimpanze or chimpanzees or chimps or cow or cows or "D melanogaster" or "dairy calf" or "dairy calves" or deer or dog or dogs or donkey or donkeys or drosophila or "Drosophila melanogaster" or duck or duckling or ducklings or ducks or equid or equids or equine or equines or feline or felines or ferret or ferrets or finch or finches or fish or flatworm or flatworms or fox or foxes or frog or frogs or "fruit flies" or "fruit fly" or "G mellonella" or "Galleria mellonella" or geese or gerbil or gerbils or goat or goats or goose or gorilla or gorillas or hamster or hamsters or hare or hares or heifer or heifers or horse or horses or insect or insects or jellyfish or kangaroo or kangaroos or kitten or kittens or lagomorph or lagomorphs or lamb or lambs or llama or llamas or macaque or macaques or macaw or macaws or marmoset or marmosets or mice or minipig or minipigs or mink or minks or monkey or monkeys or mouse or mule or mules or nematode or nematodes or octopus or octopuses or orangutan or "orang-utan" or orangutans or "orang-utans" or oxen or parrot or parrots or pig or pigeon or pigeons or piglet or piglets or pigs or porcine or primate or primates or quail or rabbit or rabbits or rat or rats or reptile or reptiles or rodent or rodents or ruminant or ruminants or salmon or sheep or shrimp or slug or slugs or swine or tamarin or tamarins or toad or toads or trout or urchin or urchins or vole or voles or waxworm or waxworms or worm or worms or xenopus or "zebra fish" or zebrafish) not (human or humans)).mp. | 8077568 |
| 62 | 59 not (60 or 61) | 1679 |
| 63 | limit 62 to (editorial or erratum or letter or note or addresses or autobiography or bibliography or biography or blogs or comment or dictionary or directory or interactive tutorial or interview or lectures or legal cases or legislation or news or newspaper article or overall or patient education handout or periodical index or portraits or published erratum or video-audio media or webcasts) [Limit not valid in Embase,CDSR,Ovid MEDLINE(R),Ovid MEDLINE(R) Daily Update,Ovid MEDLINE(R) In-Process,Ovid MEDLINE(R) Publisher; records were retained] | 97 |
| 64 | from 63 keep 1-95 | 95 |
| 65 | 62 not 64 | 1584 |
| 66 | remove duplicates from 65 | 1305 |

Scopus

1. TITLE-ABS-KEY("alibert bazin disease" OR "Andrews disease" OR "ankylating spondylitis" OR "ankylopoietic spondylarthritis" OR "ankylopoietic spondylitis" OR "ankylosing spine" OR "ankylosing spondilitis" OR "Ankylosing Spondylarthritides" OR "Ankylosing Spondylarthritis" OR "ankylosing spondylarthrosis" OR "Ankylosing Spondylitis" OR "Ankylosing Spondyloarthritides" OR "Ankylosing Spondyloarthritis" OR "ankylosis spondylitis" OR "ankylotic spondylitis" OR "arthritic psoriases" OR "arthritic psoriasis" OR "arthritis deformans" OR "arthritis psoriatica" OR "arthropathic psoriasis" OR "arthrosis deformans" OR "auto immune disease*" OR "auto immunologic disease*" OR "autoaggressive disease*" OR "autoantibody disease*" OR "autoimmune disease*" OR "autoimmune disorder*" OR "auto-immune disorder*" OR "autoimmune disturbance*" OR "autoimmune pathology" OR "autoimmuno disease*" OR "autoimmunologic disease*" OR "beauvais disease" OR "Bechterew Disease" OR "bechterew syndrome" OR "Bechterews Disease" OR "bekhterev disease" OR "bekhterevs disease" OR "chronic articular rheumatism" OR "chronic polyarthritis" OR "chronic progressive poly arthritis" OR "chronic progressive polyarthritis" OR "cleron disease" OR "colitis ulcerativa" OR "colitis ulcerosa" OR "crohn disease*" OR "crohns disease*" OR enteritis OR "Felty syndrome" OR "Feltys syndrome" OR "fereol node" OR "inflammatory arthritis" OR "inflammatory bowel disease*" OR "inflammatory enteropath*" OR "marie strumpell spondylitis" OR "Marie-Struempell Disease" OR "Marie-Struempells Disease" OR "marie-strumpell spondylitis" OR "meynet node" OR "morbus bechterew" OR "morbus crohn" OR "mucosal colitis" OR "palmar plantar bacterid" OR "palmoplantar psoriases" OR "palmoplantar psoriasis" OR "palmoplantar pustuloses" OR "palmoplantar pustulosis" OR "palmoplantaris pustuloses" OR "palmoplantaris pustulosis" OR parapsoriases OR parapsoriasis OR "peripheral pustuloses" OR "peripheral pustulosis" OR "plantar palmar bacterid" OR psoriases OR "psoriasiform dermatitis" OR "psoriasiform dermatoses" OR "psoriasiform dermatosis" OR "psoriasiform lesion*" OR "psoriasiform rash" OR "psoriasiform skin rash" OR psoriasis OR "psoriasis arthropathica" OR "psoriasis pustulosa arthropathica" OR "psoriatic arthritis" OR "psoriatic arthropath*" OR "psoriatic epidermis" OR "psoriatic polyarthritis" OR "psoriatic rheumatism" OR "psoriatic rheumatoid arthritis" OR "psoriatic skin" OR "regional enterocolitis" OR rheumarthritis OR "rheumatic arthritis" OR "rheumatic fever nodule" OR "rheumatic nodule" OR "rheumatic polyarthritis" OR "rheumatic skin disorder" OR "rheumatoid arthritis" OR "rheumatoid nodule" OR "Rheumatoid Spondylitis" OR "spinal ankylosis" OR "spine ankylosis" OR "Spondylarthritis Ankylopoietica" OR "spondylarthritis ankylosans" OR spondylarthropath* OR "spondylarthrosis ankylopoietica" OR "spondylitis ankylopoetica" OR "Spondylitis Ankylopoietica" OR "Spondyloarthritis Ankylopoietica" OR spondyloarthropath* OR "Still disease" OR "Stills disease" OR "ulcerative colitis" OR "ulcerative colorectitis" OR "ulcerative procto colitis" OR "ulcerative proctocolitis" OR "ulcerous colitis" OR "vertebral ankylosis")
2. TITLE-ABS-KEY(abatacept OR adalimumab OR alefacept OR antegren OR antiTNF OR "anti-TNF" OR "antitumor necrosis factor" OR "anti-tumor necrosis factor" OR "antitumour necrosis factor" OR "anti-tumour necrosis factor" OR avakine OR biologic OR biologics OR brodalumab OR "certolizumab pegol" OR cimzia OR "cnto 1275" OR cnto1275 OR etanercept OR golimumab OR humira OR inflectra OR infliximab OR ixekizumab OR "ldp 02" OR ldp02 OR "mln 0002" OR "mln 02" OR mln0002 OR mln02 OR "monoclonal antibody D2E7" OR "pegylated tumor necrosis factor" OR remicade OR remsima OR revellex OR rituximab OR secukinumab OR simponi OR stelara OR tocilizumab OR tofacitinib OR trudexa OR tysabri OR ustekinumab)
3. TITLE-ABS-KEY(adipos* OR antiobesity OR "anti-obesity" OR BMI OR "body ban mass" OR "body mass ind*" OR "body mass index" OR "body weight" OR bodyweight OR obes* OR obesitas OR "over eat*" OR "over fed" OR "over feed*" OR "over weight" OR overeat* OR overfed OR overfeed* OR "overload syndrom*" OR overweight OR "quetelet index" OR "quetelets index" OR "skinfold thickness" OR weight OR "weight cycling")
4. TITLE-ABS-KEY((meta W/1 analys*) OR (systematic* W/3 review*) OR cohort* OR "longitudinal study" OR "longitudinal survey" OR "longitudinal analysis" OR "longitudinal evaluation" OR ((retrospective OR "ex post facto") W/3 (study OR survey OR analysis OR design)) OR "prospective study" OR "prospective survey" OR "prospective analysis" OR (("follow-up" or followup) W/1 (stud* or survey or analysis)))
5. 1 and 2 and 3 and 4
6. TITLE-ABS-KEY(newborn* or neonat* or infant* or toddler* or child* or adolescent* or paediatric* or pediatric* or girl or girls or boy or boys or teen or teens or teenager* or preschooler* or "pre-schooler*" or preteen or preteens or "pre-teen" or "pre-teens" or youth or youths) AND NOT TITLE-ABS-KEY(adult or adults or "middle age" or "middle aged" or elderly or geriatric*)
7. 5 and not 6
8. TITLE-ABS-KEY((alpaca OR alpacas OR amphibian OR amphibians OR animal OR animals OR antelope OR armadillo OR armadillos OR avian OR baboon OR baboons OR beagle OR beagles OR bee OR bees OR bird OR birds OR bison OR bovine OR buffalo OR buffaloes OR buffalos OR "c elegans" OR "Caenorhabditis elegans" OR camel OR camels OR canine OR canines OR carp OR cats OR cattle OR chick OR chicken OR chickens OR chicks OR chimp OR chimpanze OR chimpanzees OR chimps OR cow OR cows OR "D melanogaster" OR "dairy calf" OR "dairy calves" OR deer OR dog OR dogs OR donkey OR donkeys OR drosophila OR "Drosophila melanogaster" OR duck OR duckling OR ducklings OR ducks OR equid OR equids OR equine OR equines OR feline OR felines OR ferret OR ferrets OR finch OR finches OR fish OR flatworm OR flatworms OR fox OR foxes OR frog OR frogs OR "fruit flies" OR "fruit fly" OR "G mellonella" OR "Galleria mellonella" OR geese OR gerbil OR gerbils OR goat OR goats OR goose OR gorilla OR gorillas OR hamster OR hamsters OR hare OR hares OR heifer OR heifers OR horse OR horses OR insect OR insects OR jellyfish OR kangaroo OR kangaroos OR kitten OR kittens OR lagomorph OR lagomorphs OR lamb OR lambs OR llama OR llamas OR macaque OR macaques OR macaw OR macaws OR marmoset OR marmosets OR mice OR minipig OR minipigs OR mink OR minks OR monkey OR monkeys OR mouse OR mule OR mules OR nematode OR nematodes OR octopus OR octopuses OR orangutan OR "orang-utan" OR orangutans OR "orang-utans" OR oxen OR parrot OR parrots OR pig OR pigeon OR pigeons OR piglet OR piglets OR pigs OR porcine OR primate OR primates OR quail OR rabbit OR rabbits OR rat OR rats OR reptile OR reptiles OR rodent OR rodents OR ruminant OR ruminants OR salmon OR sheep OR shrimp OR slug OR slugs OR swine OR tamarin OR tamarins OR toad OR toads OR trout OR urchin OR urchins OR vole OR voles OR waxworm OR waxworms OR worm OR worms OR xenopus OR "zebra fish" OR zebrafish) AND NOT (human OR humans))
9. 7 and not 8
10. DOCTYPE(le) OR DOCTYPE(ed) OR DOCTYPE(bk) OR DOCTYPE(er) OR DOCTYPE(no) OR DOCTYPE(sh)
11. 9 and not 10
12. PMID(0*) OR PMID(1*) OR PMID(2*) OR PMID(3*) OR PMID(4*) OR PMID(5*) OR PMID(6*) OR PMID(7*) OR PMID(8*) OR PMID(9*)
13. 11 and not 12

Web of Science

1. **TOPIC:** (("alibert bazin disease" OR "Andrews disease" OR "ankylating spondylitis" OR "ankylopoietic spondylarthritis" OR "ankylopoietic spondylitis" OR "ankylosing spine" OR "ankylosing spondilitis" OR "Ankylosing Spondylarthritides" OR "Ankylosing Spondylarthritis" OR "ankylosing spondylarthrosis" OR "Ankylosing Spondylitis" OR "Ankylosing Spondyloarthritides" OR "Ankylosing Spondyloarthritis" OR "ankylosis spondylitis" OR "ankylotic spondylitis" OR "arthritic psoriases" OR "arthritic psoriasis" OR "arthritis deformans" OR "arthritis psoriatica" OR "arthropathic psoriasis" OR "arthrosis deformans" OR "auto immune disease*" OR "auto immunologic disease*" OR "autoaggressive disease*" OR "autoantibody disease*" OR "autoimmune disease*" OR "autoimmune disorder*" OR "auto-immune disorder*" OR "autoimmune disturbance*" OR "autoimmune pathology" OR "autoimmuno disease*" OR "autoimmunologic disease*" OR "beauvais disease" OR "Bechterew Disease" OR "bechterew syndrome" OR "Bechterews Disease" OR "bekhterev disease" OR "bekhterevs disease" OR "chronic articular rheumatism" OR "chronic polyarthritis" OR "chronic progressive poly arthritis" OR "chronic progressive polyarthritis" OR "cleron disease" OR "colitis ulcerativa" OR "colitis ulcerosa" OR "crohn disease*" OR "crohns disease*" OR enteritis OR "Felty syndrome" OR "Feltys syndrome" OR "fereol node" OR "inflammatory arthritis" OR "inflammatory bowel disease*" OR "inflammatory enteropath*" OR "marie strumpell spondylitis" OR "Marie-Struempell Disease" OR "Marie-Struempells Disease" OR "marie-strumpell spondylitis" OR "meynet node" OR "morbus bechterew" OR "morbus crohn" OR "mucosal colitis" OR "palmar plantar bacterid" OR "palmoplantar psoriases" OR "palmoplantar psoriasis" OR "palmoplantar pustuloses" OR "palmoplantar pustulosis" OR "palmoplantaris pustuloses" OR "palmoplantaris pustulosis" OR parapsoriases OR parapsoriasis OR "peripheral pustuloses" OR "peripheral pustulosis" OR "plantar palmar bacterid" OR psoriases OR "psoriasiform dermatitis" OR "psoriasiform dermatoses" OR "psoriasiform dermatosis" OR "psoriasiform lesion*" OR "psoriasiform rash" OR "psoriasiform skin rash" OR psoriasis OR "psoriasis arthropathica" OR "psoriasis pustulosa arthropathica" OR "psoriatic arthritis" OR "psoriatic arthropath*" OR "psoriatic epidermis" OR "psoriatic polyarthritis" OR "psoriatic rheumatism" OR "psoriatic rheumatoid arthritis" OR "psoriatic skin" OR "regional enterocolitis" OR rheumarthritis OR "rheumatic arthritis" OR "rheumatic fever nodule" OR "rheumatic nodule" OR "rheumatic polyarthritis" OR "rheumatic skin disorder" OR "rheumatoid arthritis" OR "rheumatoid nodule" OR "Rheumatoid Spondylitis" OR "spinal ankylosis" OR "spine ankylosis" OR "Spondylarthritis Ankylopoietica" OR "spondylarthritis ankylosans" OR spondylarthropath* OR "spondylarthrosis ankylopoietica" OR "spondylitis ankylopoetica" OR "Spondylitis Ankylopoietica" OR "Spondyloarthritis Ankylopoietica" OR spondyloarthropath* OR "Still disease" OR "Stills disease" OR "ulcerative colitis" OR "ulcerative colorectitis" OR "ulcerative procto colitis" OR "ulcerative proctocolitis" OR "ulcerous colitis" OR "vertebral ankylosis")) *AND* **TOPIC:** ((abatacept OR adalimumab OR alefacept OR antegren OR antiTNF OR "anti-TNF" OR "antitumor necrosis factor" OR "anti-tumor necrosis factor" OR "antitumour necrosis factor" OR "anti-tumour necrosis factor" OR avakine OR biologic OR biologics OR brodalumab OR "certolizumab pegol" OR cimzia OR "cnto 1275" OR cnto1275 OR etanercept OR golimumab OR humira OR inflectra OR infliximab OR ixekizumab OR "ldp 02" OR ldp02 OR "mln 0002" OR "mln 02" OR mln0002 OR mln02 OR "monoclonal antibody D2E7" OR "pegylated tumor necrosis factor" OR remicade OR remsima OR revellex OR rituximab OR secukinumab OR simponi OR stelara OR tocilizumab OR tofacitinib OR trudexa OR tysabri OR ustekinumab)) *AND* **TOPIC:** ((adipos* OR antiobesity OR "anti-obesity" OR BMI OR "body ban mass" OR "body mass ind*" OR "body mass index" OR "body weight" OR bodyweight OR obes* OR obesitas OR "over eat*" OR "over fed" OR "over feed*" OR "over weight" OR overeat* OR overfed OR overfeed* OR "overload syndrom*" OR overweight OR "quetelet index" OR "quetelets index" OR "skinfold thickness" OR weight OR "weight cycling")) *AND* **TOPIC:** (((meta NEAR/1 analys*) OR (systematic* NEAR/3 review*) OR cohort* OR "longitudinal study" OR "longitudinal survey" OR "longitudinal analysis" OR "longitudinal evaluation" OR ((retrospective OR "ex post facto") NEAR/3 (study OR survey OR analysis OR design)) OR "prospective study" OR "prospective survey" OR "prospective analysis" OR (("follow-up" or followup) NEAR/1 (stud* or survey or analysis)))) *AND* **DOCUMENT TYPES:** (Article OR Abstract of Published Item OR Meeting Abstract OR Proceedings Paper OR Review) Indexes=SCI-EXPANDED, ESCI Timespan=All years
2. TS=((alpaca OR alpacas OR amphibian OR amphibians OR animal OR animals OR antelope OR armadillo OR armadillos OR avian OR baboon OR baboons OR beagle OR beagles OR bee OR bees OR bird OR birds OR bison OR bovine OR buffalo OR buffaloes OR buffalos OR "c elegans" OR "Caenorhabditis elegans" OR camel OR camels OR canine OR canines OR carp OR cats OR cattle OR chick OR chicken OR chickens OR chicks OR chimp OR chimpanze OR chimpanzees OR chimps OR cow OR cows OR "D melanogaster" OR "dairy calf" OR "dairy calves" OR deer OR dog OR dogs OR donkey OR donkeys OR drosophila OR "Drosophila melanogaster" OR duck OR duckling OR ducklings OR ducks OR equid OR equids OR equine OR equines OR feline OR felines OR ferret OR ferrets OR finch OR finches OR fish OR flatworm OR flatworms OR fox OR foxes OR frog OR frogs OR "fruit flies" OR "fruit fly" OR "G mellonella" OR "Galleria mellonella" OR geese OR gerbil OR gerbils OR goat OR goats OR goose OR gorilla OR gorillas OR hamster OR hamsters OR hare OR hares OR heifer OR heifers OR horse OR horses OR insect OR insects OR jellyfish OR kangaroo OR kangaroos OR kitten OR kittens OR lagomorph OR lagomorphs OR lamb OR lambs OR llama OR llamas OR macaque OR macaques OR macaw OR macaws OR marmoset OR marmosets OR mice OR minipig OR minipigs OR mink OR minks OR monkey OR monkeys OR mouse OR mule OR mules OR nematode OR nematodes OR octopus OR octopuses OR orangutan OR "orang-utan" OR orangutans OR "orang-utans" OR oxen OR parrot OR parrots OR pig OR pigeon OR pigeons OR piglet OR piglets OR pigs OR porcine OR primate OR primates OR quail OR rabbit OR rabbits OR rat OR rats OR reptile OR reptiles OR rodent OR rodents OR ruminant OR ruminants OR salmon OR sheep OR shrimp OR slug OR slugs OR swine OR tamarin OR tamarins OR toad OR toads OR trout OR urchin OR urchins OR vole OR voles OR waxworm OR waxworms OR worm OR worms OR xenopus OR "zebra fish" OR zebrafish) NOT (human OR humans))
3. 1 NOT 2
4. PMID=(0* or 1* or 2* or 3* or 4* or 5* or 6* or 7* or 8* or 9*)
5. 3 NOT 4
