# Supplementary material for: Obesity and response to anti-tumor necrosis factor-α agents in patients with select immune-mediated inflammatory diseases: A systematic review and meta-analysis
Source: PLoS One. 2018 May 17;13(5):e0195123. doi: 10.1371/journal.pone.0195123 (PMC5957395; doi:10.1371/journal.pone.0195123)
Supplement: S3 Text — (DOCX) [file pone.0195123.s003.docx]

**S3 Text. Actual Search Strategy – Randomized controlled trials**

Ovid

Database(s): Embase 1988 to 2017 Week 03, EBM Reviews - Cochrane Central Register of Controlled Trials November 2016, Epub Ahead of Print, In-Process & Other Non-Indexed Citations, Ovid MEDLINE(R) Daily and Ovid MEDLINE(R) 1946 to Present
Search Strategy:

| **#** | **Searches** | **Results** |
| --- | --- | --- |
| 1 | Autoimmune Diseases/ | 76837 |
| 2 | ("auto immune disease*" or "auto immunologic disease*" or "autoaggressive disease*" or "autoantibody disease*" or "autoimmune disease*" or "autoimmune disorder*" or "auto-immune disorder*" or "autoimmune disturbance*" or "autoimmune pathology" or "autoimmuno disease*" or "autoimmunologic disease*").mp. | 215545 |
| 3 | exp Arthritis, Rheumatoid/ | 266798 |
| 4 | ("arthritis deformans" or "arthrosis deformans" or "beauvais disease" or "chronic articular rheumatism" or "chronic polyarthritis" or "chronic progressive poly arthritis" or "chronic progressive polyarthritis" or "Felty syndrome" or "Feltys syndrome" or "fereol node" or "inflammatory arthritis" or "meynet node" or rheumarthritis or "rheumatic arthritis" or "rheumatic fever nodule" or "rheumatic nodule" or "rheumatic polyarthritis" or "rheumatic skin disorder" or "rheumatoid arthritis" or "rheumatoid nodule" or "Still disease" or "Stills disease").mp. | 277690 |
| 5 | exp Inflammatory Bowel Diseases/ | 182491 |
| 6 | enteritis/ | 60703 |
| 7 | exp Crohn disease/ | 106327 |
| 8 | exp ulcerative colitis/ | 86691 |
| 9 | ("inflammatory bowel disease*" or "ulcerative colitis" or "crohn disease*" or "crohns disease*" or enteritis or "inflammatory enteropath*" or "cleron disease" or "morbus crohn" or "regional enterocolitis" or "colitis ulcerativa" or "colitis ulcerosa" or "mucosal colitis" or "ulcerous colitis" or "ulcerative colorectitis" or "ulcerative procto colitis" or "ulcerative proctocolitis").tw. | 212592 |
| 10 | exp Psoriasis/ | 89031 |
| 11 | ("Andrews disease" or "arthritic psoriases" or "arthritic psoriasis" or "palmar plantar bacterid" or "palmoplantar psoriases" or "palmoplantar psoriasis" or "palmoplantar pustuloses" or "palmoplantar pustulosis" or "palmoplantaris pustuloses" or "palmoplantaris pustulosis" or parapsoriases or parapsoriasis or "peripheral pustuloses" or "peripheral pustulosis" or "plantar palmar bacterid" or psoriases or "psoriasiform dermatitis" or "psoriasiform dermatoses" or "psoriasiform dermatosis" or "psoriasiform lesion*" or "psoriasiform rash" or "psoriasiform skin rash" or psoriasis or "psoriasis arthropathica" or "psoriatic arthritis" or "psoriatic arthropath*" or "psoriatic epidermis" or "psoriatic skin" or "pustular bacterid" or "pustulosis of palms and soles" or "pustulosis palmaris" or "pustulosis palmaris et plantaris" or "pustulosis palmoplantaris" or "pustulosis plantaris" or "recalcitrant pustular eruption*" or "willan lepra").mp. | 114797 |
| 12 | 10 or 11 | 115514 |
| 13 | exp Arthritis, Psoriatic/ | 21330 |
| 14 | ("alibert bazin disease" or "arthritic psoriasis" or "arthritis psoriatica" or "arthropathic psoriasis" or "psoriasis arthropathica" or "psoriasis pustulosa arthropathica" or "psoriatic arthritis" or "psoriatic arthropath*" or "psoriatic polyarthritis" or "psoriatic rheumatism" or "psoriatic rheumatoid arthritis").mp. | 24976 |
| 15 | exp Spondylarthropathies/ | 29777 |
| 16 | ("bechterew syndrome" or "marie strumpell spondylitis" or "marie-strumpell spondylitis" or spondylarthropath* or spondyloarthropath*).mp. | 10558 |
| 17 | exp Spondylitis, Ankylosing/ | 35198 |
| 18 | ("ankylating spondylitis" or "ankylopoietic spondylarthritis" or "ankylopoietic spondylitis" or "ankylosing spine" or "ankylosing spondilitis" or "Ankylosing Spondylarthritides" or "Ankylosing Spondylarthritis" or "ankylosing spondylarthrosis" or "Ankylosing Spondylitis" or "Ankylosing Spondyloarthritides" or "Ankylosing Spondyloarthritis" or "ankylosis spondylitis" or "ankylotic spondylitis" or "Bechterew Disease" or "Bechterews Disease" or "bekhterev disease" or "bekhterevs disease" or "Marie-Struempell Disease" or "Marie-Struempells Disease" or "morbus bechterew" or "Rheumatoid Spondylitis" or "spinal ankylosis" or "spine ankylosis" or "Spondylarthritis Ankylopoietica" or "spondylarthritis ankylosans" or "spondylarthrosis ankylopoietica" or "spondylitis ankylopoetica" or "Spondylitis Ankylopoietica" or "Spondyloarthritis Ankylopoietica" or "vertebral ankylosis").mp. | 36994 |
| 19 | or/1-18 | 868505 |
| 20 | exp infliximab/ | 48901 |
| 21 | exp adalimumab/ | 27711 |
| 22 | exp certolizumab pegol/ | 4912 |
| 23 | exp golimumab/ | 4204 |
| 24 | exp etanercept/ | 30845 |
| 25 | exp abatacept/ | 9441 |
| 26 | exp tocilizumab/ | 6618 |
| 27 | exp rituximab/ | 70403 |
| 28 | exp tofacitinib/ | 1781 |
| 29 | exp ustekinumab/ | 3963 |
| 30 | exp secukinumab/ | 1153 |
| 31 | exp ixekizumab/ | 560 |
| 32 | exp brodalumab/ | 507 |
| 33 | exp alefacept/ | 1703 |
| 34 | (abatacept or adalimumab or alefacept or antegren or antiTNF or "anti-TNF" or "antitumor necrosis factor" or "anti-tumor necrosis factor" or "antitumour necrosis factor" or "anti-tumour necrosis factor" or avakine or biologic or biologics or brodalumab or "certolizumab pegol" or cimzia or "cnto 1275" or cnto1275 or etanercept or golimumab or humira or inflectra or infliximab or ixekizumab or "ldp 02" or ldp02 or "mln 0002" or "mln 02" or mln0002 or mln02 or "monoclonal antibody D2E7" or "pegylated tumor necrosis factor" or remicade or remsima or revellex or rituximab or secukinumab or simponi or stelara or tocilizumab or tofacitinib or trudexa or tysabri or ustekinumab).mp. | 310372 |
| 35 | or/20-34 | 310372 |
| 36 | 19 and 35 | 100516 |
| 37 | exp Randomized Controlled Trial/ | 966734 |
| 38 | ((randomized adj3 study) or (randomized adj3 trial) or (randomised adj3 study) or (randomised adj3 trial) or "pragmatic clinical trial").mp,pt. | 1577064 |
| 39 | 37 or 38 | 1577064 |
| 40 | 36 and 39 | 9974 |
| 41 | limit 40 to human [Limit not valid in CCTR; records were retained] | 9641 |
| 42 | (human or humans).mp. | 35480416 |
| 43 | 40 and 42 | 9640 |
| 44 | 41 or 43 | 9661 |
| 45 | limit 44 to "all adult (19 plus years)" [Limit not valid in Embase,CCTR; records were retained] | 9306 |
| 46 | limit 45 to (adult <18 to 64 years> or aged <65+ years>) [Limit not valid in CCTR,Ovid MEDLINE(R),Ovid MEDLINE(R) Daily Update,Ovid MEDLINE(R) In-Process,Ovid MEDLINE(R) Publisher; records were retained] | 5236 |
| 47 | (adult or adults or "middle age" or "middle aged" or aged or elderly or geriatric* or "old adult*" or "old people" or "old person*" or "older adult*" or "older people" or "older person*" or "very old").mp. | 14623337 |
| 48 | 44 and 47 | 4786 |
| 49 | 46 or 48 | 5443 |
| 50 | limit 49 to (editorial or erratum or letter or note or addresses or autobiography or bibliography or biography or blogs or comment or dictionary or directory or interactive tutorial or interview or lectures or legal cases or legislation or news or newspaper article or overall or patient education handout or periodical index or portraits or published erratum or video-audio media or webcasts) [Limit not valid in Embase,CCTR,Ovid MEDLINE(R),Ovid MEDLINE(R) Daily Update,Ovid MEDLINE(R) In-Process,Ovid MEDLINE(R) Publisher; records were retained] | 47 |
| 51 | 49 not 50 | 5396 |
| 52 | remove duplicates from 51 | 3101 |

Scopus

1. TITLE-ABS-KEY("alibert bazin disease" OR "Andrews disease" OR "ankylating spondylitis" OR "ankylopoietic spondylarthritis" OR "ankylopoietic spondylitis" OR "ankylosing spine" OR "ankylosing spondilitis" OR "Ankylosing Spondylarthritides" OR "Ankylosing Spondylarthritis" OR "ankylosing spondylarthrosis" OR "Ankylosing Spondylitis" OR "Ankylosing Spondyloarthritides" OR "Ankylosing Spondyloarthritis" OR "ankylosis spondylitis" OR "ankylotic spondylitis" OR "arthritic psoriases" OR "arthritic psoriasis" OR "arthritis deformans" OR "arthritis psoriatica" OR "arthropathic psoriasis" OR "arthrosis deformans" OR "auto immune disease*" OR "auto immunologic disease*" OR "autoaggressive disease*" OR "autoantibody disease*" OR "autoimmune disease*" OR "autoimmune disorder*" OR "auto-immune disorder*" OR "autoimmune disturbance*" OR "autoimmune pathology" OR "autoimmuno disease*" OR "autoimmunologic disease*" OR "beauvais disease" OR "Bechterew Disease" OR "bechterew syndrome" OR "Bechterews Disease" OR "bekhterev disease" OR "bekhterevs disease" OR "chronic articular rheumatism" OR "chronic polyarthritis" OR "chronic progressive poly arthritis" OR "chronic progressive polyarthritis" OR "cleron disease" OR "colitis ulcerativa" OR "colitis ulcerosa" OR "crohn disease*" OR "crohns disease*" OR enteritis OR "Felty syndrome" OR "Feltys syndrome" OR "fereol node" OR "inflammatory arthritis" OR "inflammatory bowel disease*" OR "inflammatory enteropath*" OR "marie strumpell spondylitis" OR "Marie-Struempell Disease" OR "Marie-Struempells Disease" OR "marie-strumpell spondylitis" OR "meynet node" OR "morbus bechterew" OR "morbus crohn" OR "mucosal colitis" OR "palmar plantar bacterid" OR "palmoplantar psoriases" OR "palmoplantar psoriasis" OR "palmoplantar pustuloses" OR "palmoplantar pustulosis" OR "palmoplantaris pustuloses" OR "palmoplantaris pustulosis" OR parapsoriases OR parapsoriasis OR "peripheral pustuloses" OR "peripheral pustulosis" OR "plantar palmar bacterid" OR psoriases OR "psoriasiform dermatitis" OR "psoriasiform dermatoses" OR "psoriasiform dermatosis" OR "psoriasiform lesion*" OR "psoriasiform rash" OR "psoriasiform skin rash" OR psoriasis OR "psoriasis arthropathica" OR "psoriasis pustulosa arthropathica" OR "psoriatic arthritis" OR "psoriatic arthropath*" OR "psoriatic epidermis" OR "psoriatic polyarthritis" OR "psoriatic rheumatism" OR "psoriatic rheumatoid arthritis" OR "psoriatic skin" OR "regional enterocolitis" OR rheumarthritis OR "rheumatic arthritis" OR "rheumatic fever nodule" OR "rheumatic nodule" OR "rheumatic polyarthritis" OR "rheumatic skin disorder" OR "rheumatoid arthritis" OR "rheumatoid nodule" OR "Rheumatoid Spondylitis" OR "spinal ankylosis" OR "spine ankylosis" OR "Spondylarthritis Ankylopoietica" OR "spondylarthritis ankylosans" OR spondylarthropath* OR "spondylarthrosis ankylopoietica" OR "spondylitis ankylopoetica" OR "Spondylitis Ankylopoietica" OR "Spondyloarthritis Ankylopoietica" OR spondyloarthropath* OR "Still disease" OR "Stills disease" OR "ulcerative colitis" OR "ulcerative colorectitis" OR "ulcerative procto colitis" OR "ulcerative proctocolitis" OR "ulcerous colitis" OR "vertebral ankylosis")
2. TITLE-ABS-KEY(abatacept OR adalimumab OR alefacept OR antegren OR antiTNF OR "anti-TNF" OR "antitumor necrosis factor" OR "anti-tumor necrosis factor" OR "antitumour necrosis factor" OR "anti-tumour necrosis factor" OR avakine OR biologic OR biologics OR brodalumab OR "certolizumab pegol" OR cimzia OR "cnto 1275" OR cnto1275 OR etanercept OR golimumab OR humira OR inflectra OR infliximab OR ixekizumab OR "ldp 02" OR ldp02 OR "mln 0002" OR "mln 02" OR mln0002 OR mln02 OR "monoclonal antibody D2E7" OR "pegylated tumor necrosis factor" OR remicade OR remsima OR revellex OR rituximab OR secukinumab OR simponi OR stelara OR tocilizumab OR tofacitinib OR trudexa OR tysabri OR ustekinumab)
3. TITLE-ABS-KEY((randomized W/3 study) OR (randomized W/3 trial) OR (randomised W/3 study) OR (randomised W/3 trial) OR "pragmatic clinical trial")
4. TITLE-ABS-KEY(human or humans)
5. TITLE-ABS-KEY(adult or adults or "middle age" or "middle aged" or aged or elderly or geriatric* or "old adult*" or "old people" or "old person*" or "older adult*" or "older people" or "older person*" or "very old")
6. 1 and 2 and 3 and 4 and 5
7. DOCTYPE(le) OR DOCTYPE(ed) OR DOCTYPE(bk) OR DOCTYPE(er) OR DOCTYPE(no) OR DOCTYPE(sh)
8. 6 and not 7
9. PMID(0*) OR PMID(1*) OR PMID(2*) OR PMID(3*) OR PMID(4*) OR PMID(5*) OR PMID(6*) OR PMID(7*) OR PMID(8*) OR PMID(9*)
10. 8 and not 9

Web of Science

1. **TOPIC:** (("alibert bazin disease" OR "Andrews disease" OR "ankylating spondylitis" OR "ankylopoietic spondylarthritis" OR "ankylopoietic spondylitis" OR "ankylosing spine" OR "ankylosing spondilitis" OR "Ankylosing Spondylarthritides" OR "Ankylosing Spondylarthritis" OR "ankylosing spondylarthrosis" OR "Ankylosing Spondylitis" OR "Ankylosing Spondyloarthritides" OR "Ankylosing Spondyloarthritis" OR "ankylosis spondylitis" OR "ankylotic spondylitis" OR "arthritic psoriases" OR "arthritic psoriasis" OR "arthritis deformans" OR "arthritis psoriatica" OR "arthropathic psoriasis" OR "arthrosis deformans" OR "auto immune disease*" OR "auto immunologic disease*" OR "autoaggressive disease*" OR "autoantibody disease*" OR "autoimmune disease*" OR "autoimmune disorder*" OR "auto-immune disorder*" OR "autoimmune disturbance*" OR "autoimmune pathology" OR "autoimmuno disease*" OR "autoimmunologic disease*" OR "beauvais disease" OR "Bechterew Disease" OR "bechterew syndrome" OR "Bechterews Disease" OR "bekhterev disease" OR "bekhterevs disease" OR "chronic articular rheumatism" OR "chronic polyarthritis" OR "chronic progressive poly arthritis" OR "chronic progressive polyarthritis" OR "cleron disease" OR "colitis ulcerativa" OR "colitis ulcerosa" OR "crohn disease*" OR "crohns disease*" OR enteritis OR "Felty syndrome" OR "Feltys syndrome" OR "fereol node" OR "inflammatory arthritis" OR "inflammatory bowel disease*" OR "inflammatory enteropath*" OR "marie strumpell spondylitis" OR "Marie-Struempell Disease" OR "Marie-Struempells Disease" OR "marie-strumpell spondylitis" OR "meynet node" OR "morbus bechterew" OR "morbus crohn" OR "mucosal colitis" OR "palmar plantar bacterid" OR "palmoplantar psoriases" OR "palmoplantar psoriasis" OR "palmoplantar pustuloses" OR "palmoplantar pustulosis" OR "palmoplantaris pustuloses" OR "palmoplantaris pustulosis" OR parapsoriases OR parapsoriasis OR "peripheral pustuloses" OR "peripheral pustulosis" OR "plantar palmar bacterid" OR psoriases OR "psoriasiform dermatitis" OR "psoriasiform dermatoses" OR "psoriasiform dermatosis" OR "psoriasiform lesion*" OR "psoriasiform rash" OR "psoriasiform skin rash" OR psoriasis OR "psoriasis arthropathica" OR "psoriasis pustulosa arthropathica" OR "psoriatic arthritis" OR "psoriatic arthropath*" OR "psoriatic epidermis" OR "psoriatic polyarthritis" OR "psoriatic rheumatism" OR "psoriatic rheumatoid arthritis" OR "psoriatic skin" OR "regional enterocolitis" OR rheumarthritis OR "rheumatic arthritis" OR "rheumatic fever nodule" OR "rheumatic nodule" OR "rheumatic polyarthritis" OR "rheumatic skin disorder" OR "rheumatoid arthritis" OR "rheumatoid nodule" OR "Rheumatoid Spondylitis" OR "spinal ankylosis" OR "spine ankylosis" OR "Spondylarthritis Ankylopoietica" OR "spondylarthritis ankylosans" OR spondylarthropath* OR "spondylarthrosis ankylopoietica" OR "spondylitis ankylopoetica" OR "Spondylitis Ankylopoietica" OR "Spondyloarthritis Ankylopoietica" OR spondyloarthropath* OR "Still disease" OR "Stills disease" OR "ulcerative colitis" OR "ulcerative colorectitis" OR "ulcerative procto colitis" OR "ulcerative proctocolitis" OR "ulcerous colitis" OR "vertebral ankylosis")) *AND* **TOPIC:** ((abatacept OR adalimumab OR alefacept OR antegren OR antiTNF OR "anti-TNF" OR "antitumor necrosis factor" OR "anti-tumor necrosis factor" OR "antitumour necrosis factor" OR "anti-tumour necrosis factor" OR avakine OR biologic OR biologics OR brodalumab OR "certolizumab pegol" OR cimzia OR "cnto 1275" OR cnto1275 OR etanercept OR golimumab OR humira OR inflectra OR infliximab OR ixekizumab OR "ldp 02" OR ldp02 OR "mln 0002" OR "mln 02" OR mln0002 OR mln02 OR "monoclonal antibody D2E7" OR "pegylated tumor necrosis factor" OR remicade OR remsima OR revellex OR rituximab OR secukinumab OR simponi OR stelara OR tocilizumab OR tofacitinib OR trudexa OR tysabri OR ustekinumab)) *AND* **TOPIC:** ((randomized NEAR/3 study) OR (randomized NEAR/3 trial) OR (randomised NEAR/3 study) OR (randomised NEAR/3 trial) OR "pragmatic clinical trial") *AND* **TOPIC:** (human or humans) *AND* **TOPIC:** (adult or adults or "middle age" or "middle aged" or aged or elderly or geriatric* or "old adult*" or "old people" or "old person*" or "older adult*" or "older people" or "older person*" or "very old") *AND* **DOCUMENT TYPES:** (Article OR Abstract of Published Item OR Meeting Abstract OR Proceedings Paper) Indexes=SCI-EXPANDED, ESCI Timespan=All years
2. PMID=(0* or 1* or 2* or 3* or 4* or 5* or 6* or 7* or 8* or 9*)
3. 1 NOT 2
