# Supplementary material for: Obesity and response to anti-tumor necrosis factor-α agents in patients with select immune-mediated inflammatory diseases: A systematic review and meta-analysis
Source: PLoS One. 2018 May 17;13(5):e0195123. doi: 10.1371/journal.pone.0195123 (PMC5957395; doi:10.1371/journal.pone.0195123)
Supplement: S4 Text — (DOCX) [file pone.0195123.s004.docx]

**S4 Text. Data Abstraction**

The following data were collected from each study: (a) study characteristics: primary author, time period of study including period of recruitment and follow-up/year of publication, country of origin, study design (RCT vs. cohort; prospective vs. retrospective), study duration (timing of outcome assessment), factors pertinent to risk of bias assessment; (b) patient characteristics: IMID type, anti-TNF agent type with corresponding dosing route and schedule, age, sex, mean BMI or weight, proportion obese, co-interventions (concomitant immunomodulators such as azathioprine, 6-mercaptopurine, or methotrexate, steroid exposure); (c) exposure characteristics: categories of BMI or weight reported using hierarchy of (i) World Health Organization-defined categories of obese [BMI≥30kg/m2], overweight [BMI 25.0-29.9kg/m2], normal BMI [BMI 18.5-24.9kg/m2], (ii) study-defined BMI categories (quartiles or tertiles), (iii) study-defined weight categories (quartiles or tertiles) or (iv) above vs. below reported BMI or weight. For all analyses, normal BMI was used as reference; if not reported, then lowest BMI/weight category was used as reference; (d) outcomes studied: type of outcomes reported, using hierarchy of (i) clinical remission or major clinical response, based on validated disease activity indices (RA: Disease Activity Score (DAS)<2.6; American College of Rheumatology (ACR)50; Crohn’s disease: Crohn’s disease activity index (CDAI)<150, Harvey Bradshaw Index (HBI)<4; ulcerative colitis: Mayo clinic score (MCS)<3 or UC clinical activity index (CAI)<4; Psoriasis: Psoriasis Area and Severity Index (PASI)75; spondyloarthropathies: ASAS Response Criteria (ASAS)20); (ii) study-defined clinical remission or response, (iii) treatment modification (need for escalation of therapy and/or switching to alternative therapy and/or surgery); (e) potential confounding variables accounted for, specifically steroid exposure, and other variables adjusted for; and (f) statistical association between obesity and outcome: unadjusted and adjusted hazard ratio (HR), relative risk (RR) or odds ratio (OR) and 95% CI, and events (failure to achieve remission/response or need for treatment modification) and total number of patients in exposed (obese) and unexposed (non-obese) groups.
