# Supplementary material for: Obesity and response to anti-tumor necrosis factor-α agents in patients with select immune-mediated inflammatory diseases: A systematic review and meta-analysis
Source: PLoS One. 2018 May 17;13(5):e0195123. doi: 10.1371/journal.pone.0195123 (PMC5957395; doi:10.1371/journal.pone.0195123)
Supplement: S1 Table — (DOCX) [file pone.0195123.s005.docx]

**S1 Table.** Study-level quality assessment using the Quality In Prognosis Studies tool.

| **Study** | **Study Participation** | **Study Attrition** | **Prognostic Factor Measurement** | **Outcome Measurement** | **Study Confounding** | **Statistical Analysis and Reporting** |
| --- | --- | --- | --- | --- | --- | --- |
| Cai, 2017 | L | L | M | L | H | H |
| Kobayashi, 2016 | L | M | H | L | H | H |
| Poulin, 2014 | L | L | H | L | H | H |
| Huang, 2014 | L | L | H | L | H | H |
| Sandborn, 2012 | L | L | H | L | H | H |
| Gottlieb, 2012 | L | L | M | L | H | H |
| Bagel, 2012 | L | M | M | L | H | H |
| Smolen, 2011 | L | L | L | L | H | H |
| Sandborn, 2011 | L | L | M | L | H | H |
| Reinisch, 2011 | L | L | H | L | H | H |
| Menter, 2010 | L | L | L | L | H | M |
| Colombel, 2010 | L | L | H | L | H | H |
| Kaeley, 2016 | L | L | L | L | H | H |
| Zweegers, 2016 | M | M | L | M | L | M |
| Villarasa, 2016 | M | M | M | M | M | M |
| Vidal, 2016^a^ | H | M | M | L | M | M |
| Menter, 2016 | L | L | L | M | L | L |
| Hojgaard, 2016 | L | M | M | L | L | L |
| Guerbau, 2016^a^ | H | M | L | H | H | H |
| Chiricozzi, 2016 | M | M | L | M | H | H |
| Brown, 2016 | L | M | L | M | M | L |
| Billiet, 2016 | L | M | M | M | H | H |
| Weinblatt, 2016 | L | L | M | L | H | H |
| Warren, 2015 | H | M | L | M | L | L |
| Iannone, 2015 | M | L | L | L | L | L |
| Simone, 2014^a^ | H | M | M | L | H | H |
| Rodrigues, 2014^a^ | H | M | M | L | H | H |
| Gremese, 2014 | L | M | L | L | H | H |
| Di Lernia, 2014 | M | M | M | M | H | H |
| Costa, 2014 | L | M | H | L | H | M |
| Leo Carnerero, 2013^a^ | H | M | M | M | H | H |
| Iannone, 2013 | H | M | L | L | L | L |
| Harper, 2013 | L | M | M | H | M | M |
| Gremese, 2013 | L | M | L | L | L | L |
| Di Minno, 2013 | L | M | M | M | L | L |
| Bhalme, 2013 | M | M | L | M | H | H |
| Rosen, 2012^a^ | H | M | H | H | H | M |
| Ottaviani, 2012 | M | M | L | L | H | M |
| Horst, 2012^a^ | H | M | M | M | H | M |
| Di Renzo, 2012 | L | M | M | M | H | H |
| Click, 2012^a^ | H | M | M | H | H | H |
| Bultman, 2012 | M | L | H | M | H | H |
| Moore, 2011^a^ | H | M | M | H | H | H |
| Klaasen, 2011 | M | M | L | L | H | H |
| Abhishek, 2010 | H | M | M | L | M | L |
| Qumseya, 2009^a^ | H | M | L | M | H | H |
| Naldi, 2008 | L | M | M | L | H | M |
| Ogdie, 2016^a^ | L | M | H | L | H | M |

^a^abstract only

[Abbreviations – ‘L’=low risk of bias, “M”=moderate risk of bias, “H”=high risk of bias]

For assessing risk of bias across each domain, the following criteria were used: **(a) Study Participation** – low risk of bias if study clearly defined sampling frame, period and place of recruitment, description of population of interest as well baseline study sample, ensured adequate participation of eligible subjects and clearly reported inclusion and exclusion criteria; moderate risk of bias if all of the above, except insufficient description of inclusion and exclusion criteria and high risk of bias, if failed to clearly define sampling frame, period and place of recruitment, inadequate description of population of interest as well baseline study sample, was not able to confirm adequate participation of eligible subjects and did not report inclusion and exclusion criteria; **(b) Study Attrition** – low risk of bias if the study reported a 100% follow-up rate or <20% attrition at end of study, or in case of >20% attrition, a clear statement that patients compliant with follow up were not significantly different from those lost to follow-up; moderate risk of bias if study did not report any attrition rate or an attrition of >20% but with no description of any systematic differences between those followed and those lost to follow-up, and high-risk of bias if attrition was >20% with reported systematic differences between those followed and those lost to follow-up; **(c) Prognostic factor measurement** – low risk of bias if studies clearly described assessment of obesity based on measured values of BMI (not self-reported), used appropriate WHO-defined cut-offs (or at least 3 categories); moderate risk of bias if only 2 categories of BMI reported and high risk of bias if only 2 categories of weight reported; **(d) Outcome Measurement** – low risk of bias if study clearly and appropriately defined outcomes as clinical remission or response (based on validated disease activity index); moderate risk of bias if study used pragmatic definition of failure of therapy (escalation of therapy and/or switching to another therapy), and high-risk of bias if other definitions; **(e) Study Confounding** – low risk of bias if study reports adjusted OR with adjustment for baseline disease activity AND use of corticosteroids; moderate risk of bias if study reports adjusted OR reported for only one of the mentioned risk factors and high-risk of bias if the study reports only unadjusted analysis; **(f) Statistical Analysis and Reporting** – low risk of bias if study performed multivariate cox proportional hazard model without over-fitting; moderate risk of bias if study reports multivariate cox regression analysis instead of time to event analysis, and high-risk of bias if study just reported univariate analysis or if there is selective reporting of results
