# Supplementary figures and images for: Obesity and response to anti-tumor necrosis factor-α agents in patients with select immune-mediated inflammatory diseases: A systematic review and meta-analysis
Source: PLoS One. 2018 May 17;13(5):e0195123. doi: 10.1371/journal.pone.0195123 (PMC5957395; doi:10.1371/journal.pone.0195123)

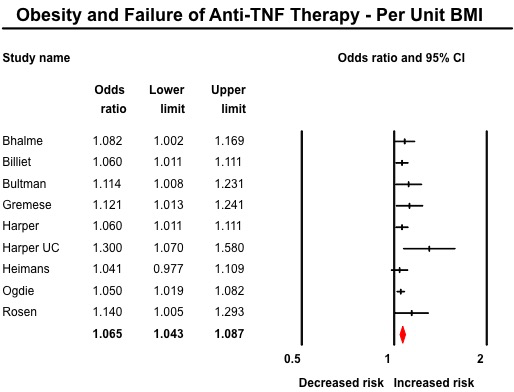

Supplement: S1 Fig — (TIFF) [file pone.0195123.s006.tiff]

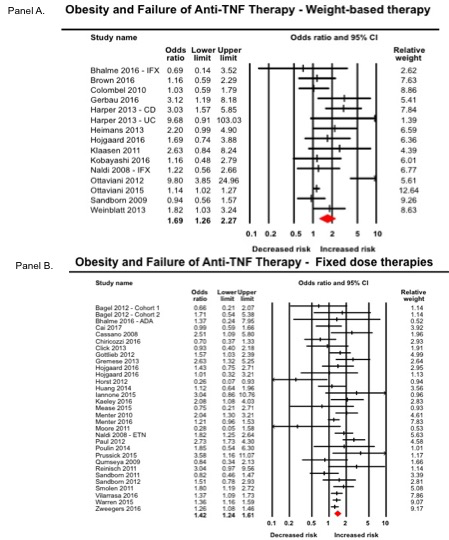

Supplement: S2 Fig — Association between obesity and response to anti-TNF therapy–Subgroup analyses based on anti-TNF dosing regimen: (A) weight-based dosing, and (B) fixed dose therapies. (TIFF) [file pone.0195123.s007.tiff]

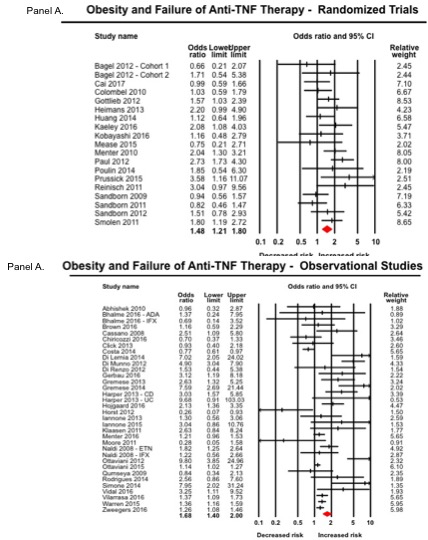

Supplement: S3 Fig — Association between obesity and response to anti-TNF therapy–Subgroup analyses based on study design: (A) RCTs, and (B) observational studies. (TIFF) [file pone.0195123.s008.tiff]

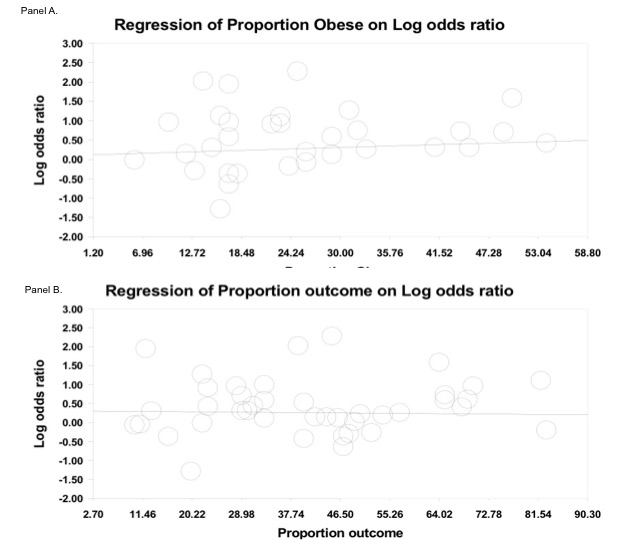

Supplement: S4 Fig — Meta-regression based on (A) prevalence of obesity, and (B) prevalence of outcome. (TIFF) [file pone.0195123.s009.tiff]

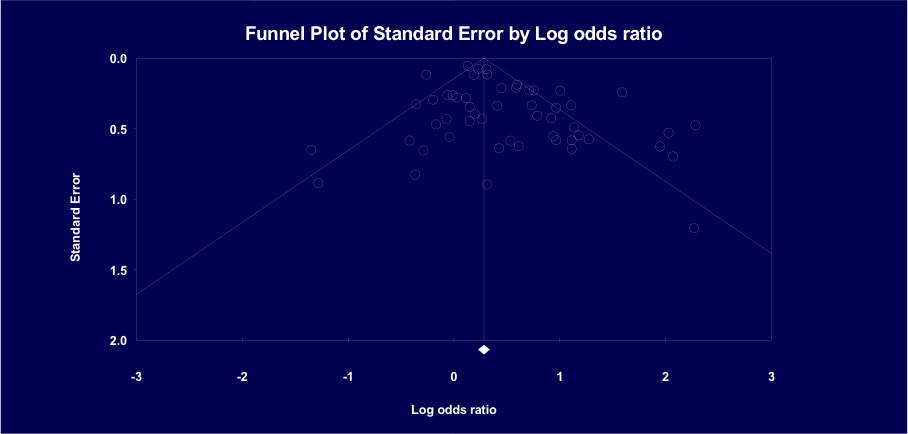

Supplement: S5 Fig — (TIFF) [file pone.0195123.s010.tiff]
